# Human-Mediated Marine Dispersal Influences the Population Structure of *Aedes aegypti* in the Philippine Archipelago

**DOI:** 10.1371/journal.pntd.0003829

**Published:** 2015-06-03

**Authors:** Eugenio Fonzi, Yukiko Higa, Arlene G. Bertuso, Kyoko Futami, Noboru Minakawa

**Affiliations:** 1 Department of Vector Ecology and Environment, Institute of Tropical Medicine, Nagasaki University, Nagasaki, Japan; 2 Department of Public Health, University of the Philippines, Manila, Philippines; Centers for Disease Control and Prevention, Puerto Rico, UNITED STATES

## Abstract

**Background:**

Dengue virus (DENV) is an extraordinary health burden on global scale, but still lacks effective vaccine. The Philippines is endemic for dengue fever, but massive employment of insecticides favored the development of resistance mutations in its major vector, *Aedes aegypti*. Alternative vector control strategies consist in releasing artificially modified mosquitos in the wild, but knowledge on their dispersal ability is necessary for a successful implementation. Despite being documented that *Ae*. *aegypti* can be passively transported for long distances, no study to date has been aimed at understanding whether human marine transportation can substantially shape the migration patterns of this mosquito. With thousands of islands connected by a dense network of ships, the Philippines is an ideal environment to fill this knowledge gap.

**Methodology/principal findings:**

Larvae of *Ae*. *aegypti* from 15 seaports in seven major islands of central-western Philippines were collected and genotyped at seven microsatellite loci. Low genetic structure and considerable gene flow was found in the area. Univariate and multivariate regression analyses suggested that anthropic factors (specifically the amount of processed cargo and human population density) can explain the observed population structure, while geographical distance was not correlated. Interestingly, cargo shipments seem to be more efficient than passenger ships in transporting *Ae*. *aegypti*. Bayesian clustering confirmed that *Ae*. *aegypti* from busy ports are more genetically similar, while populations from idle ports are relatively structured, regardless of the geographical distance that separates them.

**Conclusions/significance:**

The results confirmed the pivotal role of marine human-mediated long-range dispersal in determining the population structure of *Ae*. *aegypti*. Hopefully corroborated by further research, the present findings could assist the design of more effective vector control strategies.

## Author Summary

Dengue fever threatens the health of millions in the tropics and its causative agent, dengue virus, is mainly transmitted by the mosquito *Aedes aegypti*. To control the spread of the virus, insecticides have been abundantly used but *Ae*. *aegypti* has developed a genetic resistance to them. Currently, alternative methods are being tested wherein artificially modified mosquitos are released in the wild to interfere with the mating of natural populations. It is important then to understand how the mosquito spreads in the environment. It is known that *Ae*. *aegytpi* can be passively transported for long distances by human vehicles, but it was not clear how common this event is, especially in case of marine transportation. In population genetics, a basic assumption says that if populations frequently exchange migrants, they become genetically more similar than relatively isolated populations. We estimated the genetic similarity between *Ae*. *aegypti* collected in the Philippines from 15 seaports of different sizes and ship connectivity. The mosquitos from busy ports, even distant ones, were genetically similar, while in the small ports, even close ones, *Ae*. *aegypti* were relatively differentiated. It was also suggested that *Ae*. *aegypti*'s dispersal is affected by cargo shipments more than passenger ships.

## Introduction

*Aedes aegypti* is the major vector of dengue virus (DENV). An estimated 96 million dengue cases and 300 million asymptomatic infections were reported globally in 2010 [1]. Currently, there is no effective dengue vaccine available and the principal way to prevent the spread of the virus and the occurrence of outbreaks is to suppress the populations of vector mosquitos. Insecticide spraying has been the most common and effective method in the past decades, but massive and indiscriminate use generated a selective pressure that led to the development of insecticide-resistant strains of mosquitos. Alternative population control methods consist in the release of modified mosquitos which will interbreed with wild populations and suppress or replace it. Depending on the technique, the released individuals are sterilized, genetically engineered or carry symbiotic bacteria of the genus *Wolbachia* [2].

The dispersal ability of the vector should be taken into account when implementing any of the above-mentioned population suppression techniques. If a vector can travel long distances it can quickly and effectively spread insecticide resistance mutations, symbiotic bacteria or deleterious mutations.

The flight range of *Ae*. *aegypti* is narrow (usually less than 200m, rarely up to 500m) [3–5] and its active dispersal in the wild should be limited. However, this mosquito is believed to occasionally travel long distances by taking advantage of human (land, sea or air) vehicles [6–8]. There are reports of *Ae*. *aegypti* collected from ships right after docking [9] and the trade of used tires is suspected to be one of the main contributions to the passive dispersal of *Ae*. *aegypti* immatures, both by land and sea [10]. Recently, larvae and pupae of *Ae*. *aegypti* have been for the first time collected at Narita Airport (Tokyo, Japan). It is strongly suspected that they were transported via airplane from a foreign tropical country [11]. If *Ae*. *aegypti*'s migration flows are mainly driven by active dispersal, the genetic differentiation between populations should increase with the geographical distance; this is called Isolation By Distance (IBD) [12]. Otherwise, if passive human-mediated dispersal is pivotal, IBD should be weak or absent.

A remarkable amount of population genetics studies have been conducted on *Ae*. *aegypti* in many regions of the world. The genetic structure of this important vector mosquito has been analyzed at micro and macro geographical scales with different methods and purposes. Long-distance migration has been hypothesized and frequently related to human movements, but this association was not addressed in a quantitative way and only briefly brought up in the final discussion as a possible explanation of the results [13–15]. A few studies had their main focus on the effects of human transportation on *Ae*. *aegypti*'s dispersal; yet, only the land transportation was taken into account [16–20]. One study undertaken in French Polynesia referred to the effect of human sea transportation; however, its main purpose was to determine if the circulation of DENV strains between Polynesian islands could be related to *Ae*. *aegypti*'s migration [21]. Therefore, no study to date has been specifically aimed at quantitatively clarifying whether human-mediated long-distance marine dispersal is able to shape the genetic structure of *Ae*. *aegypti* at a macro-geographical scale.

The Philippines is an archipelago located in Southeast Asia and is endemic for dengue fever. According to the World Health Organization, the annual reported cases of dengue fever increased around 17-fold in the decade 2000–2010 [22]. Unlike other southeast Asian countries (mainly Thailand and Vietnam), very few ecological studies and no population genetics study have been conducted on *Ae*. *aegypti* in the Philippines [23–26]. A recent survey conducted by our group in the northern part of the country showed a widespread resistance to pyrethroid insecticides and a high prevalence of kdr mutations [27,28]. Furthermore, the nearly 7000 islands that make up the Philippine archipelago are connected by a dense network of cargo and passenger ships. The islands are remarkably different in size, demography and economic development; therefore, the seaports as well are strikingly different in size and commercial importance. Busy ports are more interconnected than idle ports, regardless of their geographical position. This offers a proper environment for the randomization of collection sites.

For these reasons we decided to undertake a population genetics study aimed at understanding if and how human marine transportation affects the movements of *Ae*. *aegypti* among different islands of the Philippines. Specific objectives were: (1) to analyze the genetic structure of *Ae*. *aegypti* among different islands; (2) to quantitatively determine if natural (geographical distance) or anthropic (port size and intensity of marine transportation) factors affect the observed genetic structure.

## Methods

### Sampling

**Fig 1:**
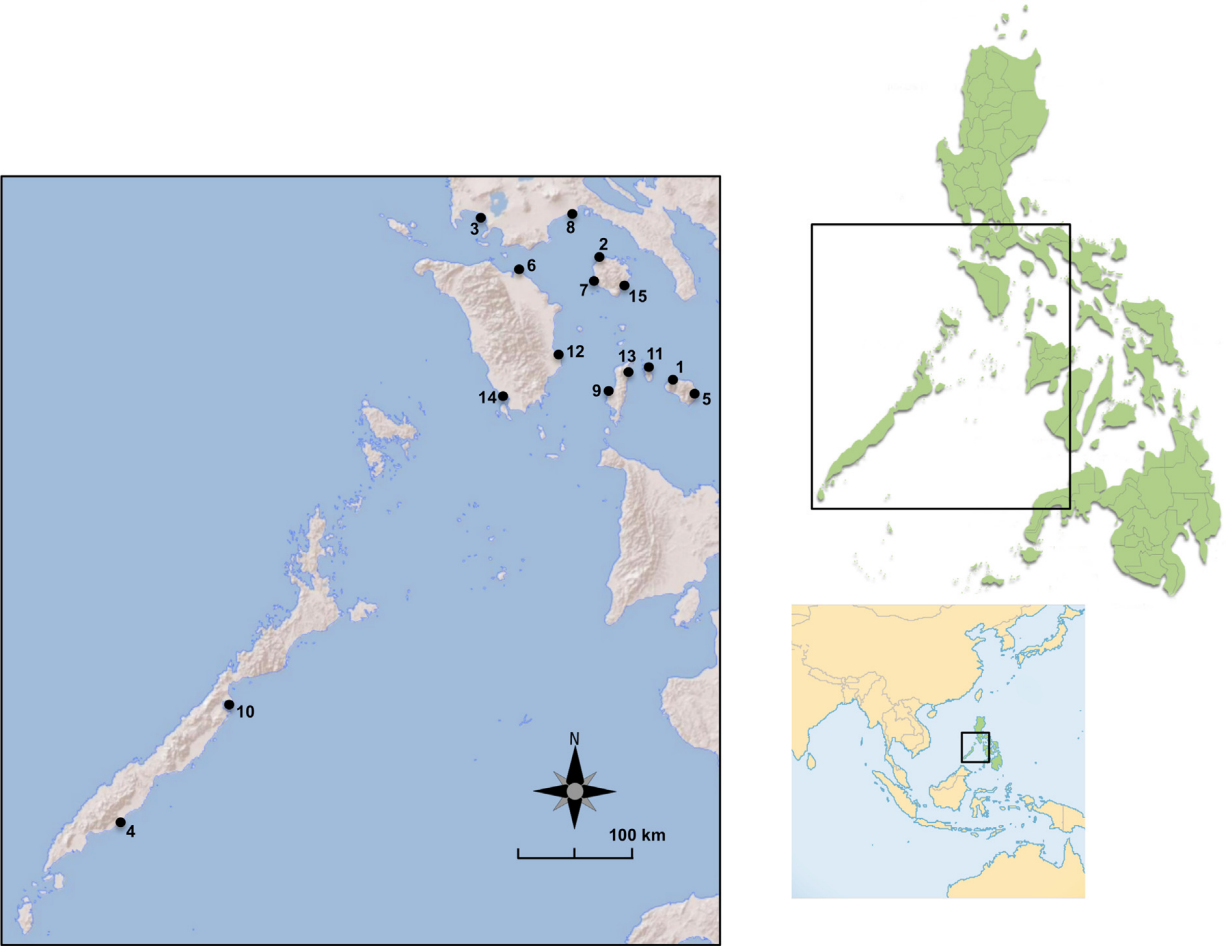
Map of the sites where *Ae*. *aegypti* were collected. For detailed populations information refer to Table 1.

In order to analyze the effect of human sea transportation on the migration of *Ae*. *aegypti*, seaport locations on several islands were sampled, as populations of mosquitos residing near the seaports are more likely to end up resting or laying eggs on the ships (either cargo or passenger). Each site corresponded to one seaport and all individuals from a site were considered as one separate population, therefore the terms "site", "population" and "port" will be used interchangeably, hereinafter. The survey was conducted between September and October 2013 in the central-western area of the Philippines (Region IV-A and IV-B). Larvae were collected from 15 ports of different levels of connectivity and development, across seven islands of different sizes and degrees of urbanization (Fig 1 and Table 1). At each site, as many water-filled containers as possible (artificial or natural) were inspected in a 1.5 km-radius area; the radius was calculated from the center of the port. In case the container was located in a private area, the inspection was conducted only with consent of the owner. Collected larvae were conserved in absolute ethanol and identified by microscopic analysis [29,30] or by PCR amplification with species-specific primers [31]. Only larvae identified as *Ae*. *aegypti* were included in the genetic study. In order to reduce the proportion of sibling individuals (which overestimate the genetic difference between populations), *Ae*. *aegypti*'s flight range and its so-called "skip oviposition" behavior were taken into account [32] and a minimum distance of 200m was kept between the containers; then, one out of five larvae were randomly selected from each container and included in the genetic analysis. For port 6 one out of six larvae were selected. In case it was not possible to keep the minimum distance of 200m, larvae from multiple containers were grouped as if collected from the same.

**Table 1:** Details of the populations studied.

Site Number^1^	Site Name	Latitude	Longitude	Containers^2^	Individuals^3^	N^4^
1	Ambulong	12°29'34.10"N	122°29'27.78"E	8/10	346	67
2	Balanacan	13°32'0.02"N	121°51'59.28"E	7/12	112	24
3	Batangas	13°45'5.68"N	121°2'36.99"E	8/9	288	56
4	Brooke's Point	8°46'16.83"N	117°50'9.97"E	4/11	235	49
5	Cajidiocan	12°22'2.06"N	122°40'41.43"E	6/11	268	53
6	Calapan	13°25'37.12"N	121°11'46.03"E	7/11	301	48
7	Cawit	13°22'55.07"N	121°49'27.23"E	6/13	60	14
8	Lucena	13°54'18.18"N	121°37'18.06"E	7/12	279	56
9	Odiongan	12°25'4.53"N	121°59'46.95"E	4/12	195	40
10	Puerto Princesa	9°44'22.93"N	118°44'30.24"E	8/14	204	43
11	Romblon	12°34'52.48"N	122°15'59.25"E	5/15	93	19
12	Roxas	12°35'37.56"N	121°31'10.47"E	5/13	140	30
13	San Agustin	12°34'3.81"N	122°8'5.02"E	4/6	324	64
14	San Jose	12°19'51.36"N	121°5'12.97"E	7/15	152	31
15	Torrijos	13°19'21.40"N	122°5'15.79"E	8/12	231	48

^1^Refer to Fig 1 for geographical location of each site.

^2^Containers inspected (positive for *Ae*. *aegypti*/total).

^3^Total *Ae*. *aegypti* collected.

^4^Individuals selected for the study.

### Genotyping

Genomic DNA was extracted from individual larvae with RedExtract-N-Amp^TM^ Tissue PCR Kit (SIGMA, USA) following the manufacturer's protocol. Seven microsatellite loci were scored for each individual. Each PCR reaction (10μl) contained 400nM of M13 fluorescent primer (FAM-labeled) [33], 200nM of M13-tailed forward primer, 200nM of reverse primer, 1X of Buffer (Mg^2+^ 2mM included), 200nM of each dNTP, 0.25U of Takara ExTaq (Takara BIO, Inc.) and 1μl of sample. Thermocycling conditions were: (5' at 94°C) for one cycle, (30'' at 94°C, 30'' at T_a_, 40'' at 72°C) for 35 cycles, (5' at 72°C) for 1 cycle and 4°C as holding T. T of annealing (T_a_) was specifically determined for each pair of primers (S1 Table). After PCR amplification, 1.5μl of product was added to 8.5μl of a GeneScan™ 500 ROX Size Standard:formamide solution (1:100), heated at 95°C for 5' and run on an Applied Biosystems 3730*xl* DNA Genetic Analyzer. The allele scoring was conducted with the GeneMapper Software 5 (Applied Biosystems).

### Marker selection

Seven microsatellite markers were selected out of 74 loci previously characterized in other studies [34–38]. After a search in the database, 33 were found to reside on different supercontigs. Preliminary PCR reactions with different T_a_ were conducted in order to find the most suitable for each primer pair (S1 Table). Trial runs on capillary electrophoresis were performed to select the markers that reliably amplified in most of the samples, with sufficient allelic variability and that could be clearly and easily scored in the software GeneMapper. Many loci were discarded because the size of some of their allele peaks were misaligned with the sizes expected from their repetitive unit size; most of those loci turned out to be located near poly-A segments or to have deletions/insertions not related to the short tandem repeat of interest.

### Data analyses

The software GENEPOP v4.2.1 [39,40] was used to compute the basic population genetic parameters. The Inbreeding Coefficients (Fis) were calculated for all loci across populations according to Weir and Cockerham [41], in order to detect any statistically significant deviation from Hardy-Weinberg Equilibrium (HWE). Each pair of loci was also tested for Linkage Disequilibrium (LD). Markow Chain parameters were set at 10,000 dememorizations, 20 batches, 5000 iterations for the former analysis and at 10,000 dememorizations, 100 batches, 5000 iterations for the latter one. GENEPOP was also used to calculate total and pairwise Fixation Indexes (Fst); P-values for pairwise Fst were computed with the software Arlequin v3.5.1.3 [42].

To test the hypothesis that human transportation influences mosquitos' dispersal, eight variables were elaborated; two were obtained from satellite images and six from official data published online by the Philippine Statistics Authority (Table 2 and S2 Table). The variable named "Distance" accounts for the geographical location of the ports. The other seven variables account for port size and degree of ship connectivity, therefore we will refer to them as Anthropic Variables (AVs) hereinafter. Two AVs ("Inhabitant" and "Density") are demographic data on the municipalities where ports are located. The variable named "Dock" expresses the physical size of a port, by measuring the perimeters of its concrete docks. The last four AVs ("Vessel", "Tonnage", "Cargo", "Passenger") measure the intensity of ship traffic at ports. The statistics for the last four AVs were incomplete and their correspondent values for ports 1, 5, 13 and 15 were missing. In order to interpolate them, four bivariate linear regressions with Inhabitant and Dock as independent variables were run. Inhabitant and Dock were chosen because of high correlation with Vessel, Tonnage, Cargo and Passenger (Pearson's r≥0.47, S3 Table). The command "lm" in the software R for MachIntosh (v3.1.1 GUI 1.65 Snow Leopard build) was used for the purpose.

**Table 2:** Definitions of the variables used to test the hypothesis that human transportation influences population structure of *Ae*. *aegypti* in the central-western Philippines.

Name	Definition
Fst	Fixation Index calculated for each pair of *Ae*. *aegypti* populations, using allelic frequencies at seven microsatellite loci (S1 Table); high Fst values indicate genetic differentiation, while low Fst indicate genetic similarity
Distance	Euclidean distance in km between each pair of ports^2^
Inhabitant^1^	Total human population of the municipality to which each port belongs^3^
Density^1^	Total human population of the municipality to which each port belongs, divided by the total area of the municipality in square kilometres^3^
Dock^1^	Total sum of the perimeters (in meters) of all the concrete docks of a port^2^
Vessel^1^	Total annual number of domestic vessels visiting a port, inbound and outbound (average from 2000 to 2011)^4^
Tonnage^1^	Total annual Gross Register Tonnage (a ship's total internal volume expressed in register tons) of the domestic vessels visiting a port, inbound and outbound (average from 2000 to 2011)^4^
Cargo^1^	Total annual domestic cargo throughput of a port, in metric tons, inbound and outbound (average from 2000 to 2011)^4^
Passenger^1^	Total annual number of passengers visiting a port, inbound and outbound (average from 2000 to 2011)^4^

^1^Originally single data for a single port, then transformed to pairwise using the formula *a+b* where *a* and *b* are single values for each port.

^2^Calculated from satellite images using the "ruler" function of Google Earth.

^3^Official data from the Philippine Statistics Authority—National Statistics Office (http://web0.psa.gov.ph/).

^4^Official data from the Philippine Ports Authority (http://www.pdosoluz.com.ph/).

The command "princomp" in R was used to run a Principal Component Analysis (PCA) on the AVs. As the data showed considerable heteroscedasticity they were log-transformed before PCA [43].

As the AVs were expressed as a single value for each port (15 values per variable), while Fst values were pairwise (105 total port-by-port pairs, excluding same-port pairs), in order to quantitatively compare them, the values for the AVs were transformed to pairwise; the formula *a+b* was used, were *a* and *b* are the corresponding value for each two ports. Pearson correlation coefficients were computed in R with the command "rcorr". To detect collinearity, the variance inflation factors (VIF) between eight predictors were computed (command "vif"). The command "lm" was used to run the multivariate linear regression models. The best model was chosen with the command "step", which performs a stepwise exclusion of each predictor and calculates the Akaike Information Criterion. Simple and partial Mantel tests were run using the online software "Isolation by distance web service" (IBDWS) [44]. One-tailed P-values were calculated with 10,000 bootstrap randomizations.

The Bayesian clustering method implemented in the software STRUCTURE v. 2.3.4 [45] was used to assign individual *Ae*. *aegypti* to genetic clusters based on the microsatellite allelic frequencies. Admixture was selected as ancestry model with LOCPRIOR function. Alpha was allowed to vary and was separately calculated for each population. A correlated allele frequency model was chosen, with lambda set to one. Ten independent replicates were run with a burn-in step of 300,000 iterations followed by 500,000 replications. Average L(K), ΔK [46] and proportions of membership (i.e. the probability of a population to belong to a certain cluster, we will hereinafter refer to it as POM) were computed in Excel. Pearson correlation coefficients between POM and principal component 1 (PC1) were computed in R with the command "cor.test".

## Results

### Sampling

An average of 211 *Ae*. *aegypti* larvae per site were collected (total = 3166). At each site between six and 15 containers were inspected and between four and eight were positive. One out of five larvae was randomly selected from each container and an average of 43 per population was genotyped (total = 642) (Table 1).

### Marker validation

The test for deviation from HWE calculated 105 Fis values [41], one for each locus-population pair (S4 Table). Nine (8.6%) showed significant departure from HWE (after Bonferroni sequential correction), eight towards a heterozygosity deficit and one towards a heterozygosity excess. However, no locus showed significant values at more than three populations.

The test for LD calculated a total of 315 values (21 locus-by-locus pairs for each population), of which 28 (8.9%) remained significant after Bonferroni sequential correction. However, no locus-by-locus pair showed significance consistently across populations. Therefore this significance was unlikely due to linkage. None of the markers was excluded from the analysis.

### Genetic structure of *Ae*. *aegypti*

For most loci between four and six alleles were detected; only AG5 had 11. The Fst for all loci and populations was 0.056, while the pairwise Fst ranged from 0.005 and 0.147; after Bonferroni correction 79/105 (75.2%) remained significant (P<0.0005) (Table 3).

**Table 3:** Fixation indexes (Fst) calculated for each pair of populations studied.

	1	2	3	4	5	6	7	8	9	10	11	12	13	14
**2**	0.060*													
**3**	0.051*	0.010												
**4**	0.119*	0.029*	0.069*											
**5**	0.099*	0.045*	0.049*	0.087*										
**6**	0.081*	0.018	0.023*	0.077*	0.040*									
**7**	0.077*	0.019	0.062*	0.083*	0.069*	0.060*								
**8**	0.056*	0.023	0.009	0.075*	0.041*	0.038*	0.073*							
**9**	0.147*	0.042*	0.051*	0.072*	0.105*	0.066*	0.131*	0.066*						
**10**	0.063*	0.021	0.016	0.057*	0.046*	0.059*	0.074*	0.022*	0.077*					
**11**	0.024	0.019	0.022	0.073*	0.070*	0.061*	0.043	0.022	0.081*	0.029				
**12**	0.047*	0.007	0.009	0.047*	0.044*	0.037*	0.034	0.017	0.079*	0.023	0.005			
**13**	0.103*	0.059*	0.049*	0.075*	0.118*	0.117*	0.086*	0.067*	0.118*	0.062*	0.051*	0.025		
**14**	0.080*	0.021	0.019	0.097*	0.031*	0.020	0.053*	0.029*	0.067*	0.029*	0.030	0.034	0.104*	
**15**	0.106*	0.039*	0.034*	0.093*	0.064*	0.061*	0.057*	0.051*	0.101*	0.035*	0.049*	0.046*	0.081*	0.023

*P<0.0005.

### Statistical analysis of factors affecting *Ae*. *aegypti*'s genetic structure

**Fig 2:**
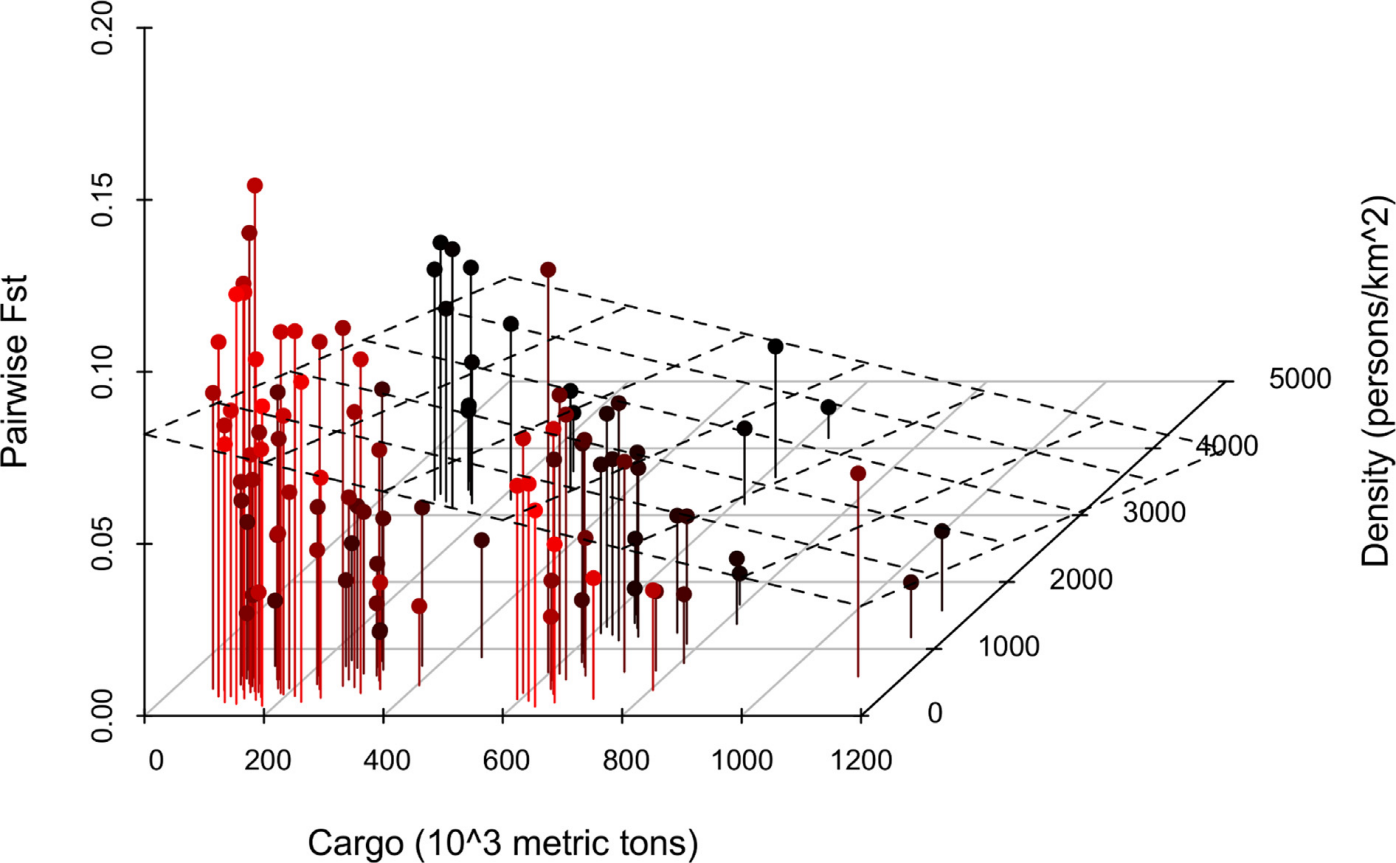
Three-dimensional scatter plot with regression plane that visualizes the effect of the variables called “Density” and “Cargo” on pairwise Fst values. Both predictors are negatively correlated to Fst and the relative regression coefficients are very low in absolute value. This means that a remarkable increase in Cargo and Density is necessary to cause a unit decrease in Fst values. The interpretation is that where cargo shipments are intense and human population is dense *Ae*. *aegypti* are more genetically similar. This suggests an influence of human transportation on mosquitos’ migration. Refer to Table 2 for detailed description of the variables.

Pearson correlation coefficient for Distance against Fst was close to zero and non-significant, while it was highly significant for all the AVs against Fst (P<0.01) (Table 4). Tonnage and Passenger were excluded from the multivariate linear regression because of high collinearity (square root of VIF>10). Distance, Inhabitant, Dock and Vessel were dropped after stepwise analysis. The multiple R^2^ was 0.24 (P<0.001). The regression coefficients for Density and Cargo indicated a negative association with pairwise Fst (P<0.001) (Table 4 and Fig 2). The square roots of VIFs are displayed in S5 Table.

**Table 4:** Results of regression models.

Predictor	Pearson's r	Multiple linear regression		
		Excluded^1^	Dropped^2^	Significant^3^
**Distance**	0.09		✗	
**Inhabitant**	**-0.44**		✗	
**Density**	**-0.34**			**-1.03e-05**
**Dock**	**-0.36**		✗	
**Vessel**	**-0.29**		✗	
**Tonnage**	**-0.3**	✗		
**Cargo**	**-0.37**			**-4.16e-08**
**Passenger**	**-0.33**	✗		

In all cases pairwise Fst is the dependent variable. Bold indicates P<0.01 for Pearson and P<0.001 for multiple linear regression.

^1^Predictors excluded before running the model because the square root of Variance Inflation Factor (VIF) was >10.

^2^Predictors included in the model, but dropped by the stepwise analysis.

^3^Regression coefficients of the predictors confirmed by the model. Refer to Table 2 for detailed description of the predictors.

**Fig 3:**
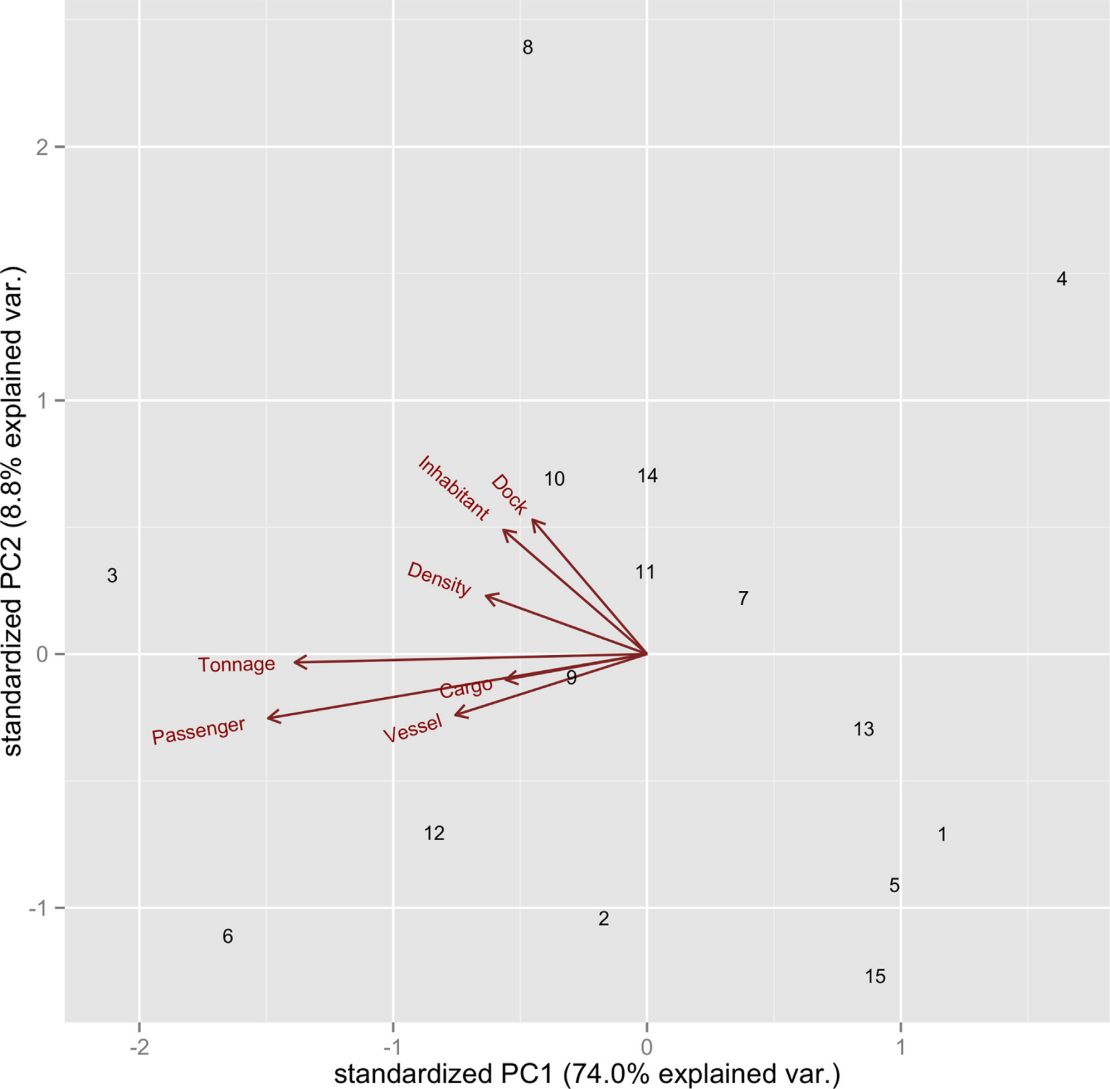
Principal component analysis used to summarize seven variables related to port size and connectivity; plot of the first two PCs. Red arrows represent the vectors of the seven variables and each point in the plot is a port (Fig 1 and Table 1). The direction of principal component 1 (PC1) is almost opposite to the variables and it explains 74% of the total variance in the dataset; therefore, ports with low values of PC1 were considered big and highly connected (and vice versa). Refer to Table 2 for a detailed description of each variable.

The PCA on the seven AVs returned a PC1 able to explain 74% of the total variance of the dataset; the directions of the vectors representing the AVs were almost opposite to the PC1 (Fig 3). It is therefore reasonable to assume that higher PC1 values indicate ports located in less developed and less connected municipalities and vice versa.

**Fig 4:**
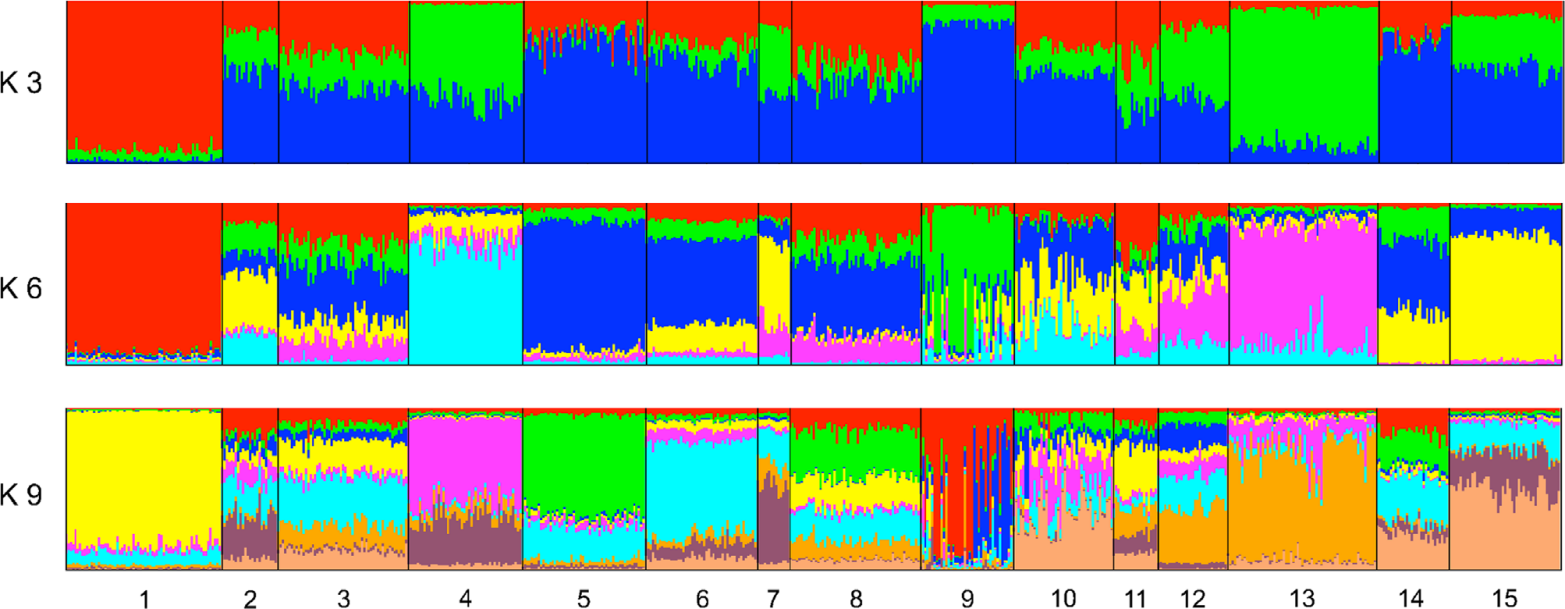
STRUCTURE bar plots. Each individual is represented by a vertical bar whose colors show the probability to be assigned to specific clusters. Populations are separated by vertical black lines and identified by the numbers at the bottom (Fig 1 and Table 1). K = number of genetic clusters. Overall, a low level of genetic structure was detected. At K6 and K9 most populations from busy ports show genetic admixture, while most populations from idle ports are genetically clustered (compare with Fig 3).

STRUCTURE analysis could detect no genetic structure unless the option LOCPRIOR was chosen. This function uses information on sampling locations to assist the clustering in case the signal of structure is too weak [47]. A considerable degree of admixture was detected and most of the individuals were assigned to several clusters with almost equal probability. Nevertheless, with increasing K some populations had increasing probability to be assigned to a specific cluster (Fig 4). Following the method proposed by Evanno et al. [46] we found peaks of ΔK at K = 3, K = 6 and K = 9 the last one being the highest (S1 Fig). The peak at K = 3 was higher than K = 6, but the bar plot of the latter was considerably more informative. The highest ΔK was at K = 9, but L(K) decreases considerably between K = 6 and K = 9 (S1 Fig) and the additional structure explained by K = 9 is not remarkable (Fig 4). Therefore, we discuss clusterings at both K = 6 and K = 9. Pearson correlation coefficients of POM regressed on PC1 scores were both high and significant (for K = 6, r = 0.76, P<0.01; for K = 9, r = 0.57, P<0.05).

## Discussion

The results suggested low genetic structure and considerable gene flow. Pairwise Fst values were generally low (0.005~0.147), in accordance with other studies conducted in Florida (0.004~0.171), Pakistan (0.013~0.17) and Southeast Asia (0.02~0.185) [17,19,48]. In the study area the migration of mosquitos was not affected by geographical distance, but increased with the intensity of human marine transportation. In our understanding, this is the first study that qualitatively and quantitatively shows how human ship transportation affects the migration of *Ae*. *aegypti*.

### Regression models

Both univariate and multivariate regression models excluded IBD. In fact, Distance/Fst Pearson's r was almost zero (0.09; non-significant) and Distance was dropped by multiple linear regression stepwise analysis (Table 4). On the other hand, Pearsons supported a negative correlation between Fst and the AVs (all r values ≤-0.3; P<0.01). The multivariate analysis confirmed the correlation only for Density and Cargo (P<0.001). The multiple linear regression coefficients were negative and very small in absolute value, therefore pairwise Fst decreases very slowly as Density and Cargo increase (Table 4); this is also evident in the scatter plot (Fig 2).

Our interpretation is that increasing cargo shipments increase the probability for *Ae*. *aegypti* to be accidentally transported, decreasing the genetic differentiation and consequently the Fst. Similarly, more densely populated municipalities have likely busier ports, with more congested ship traffic and offer more opportunities to *Ae*. *aegypti* to be transported.

The results also suggest that cargo shipments transport *Ae*. *aegypti* more effectively than passenger ships. If we consider that cargo ships (like barges) are usually bigger in size than passenger ships, they may offer a higher number of breeding sites (i.e. collections of water). Moreover, the cargo storage compartments are usually kept closed during the navigation, while passenger ships' decks are often not enclosed; this could cause more adult mosquitos to enter and being effectively transported by the cargo ships. Another hypothesis (not excluding the previous ones) is that cargo shipment routes are implemented earlier than passenger ships' routes during the development of the ports. In other words, ports of small and intermediate sizes could be connected by more cargo than passenger ships, while busiest ports operate a similar amount of the two ship types. This hypothesis is supported by the scatter plots between the four "ship connectivity" predictors (i.e. Vessel, Tonnage, Cargo and Passenger); all of them are highly correlated, but only Cargo shows a non-linear relation with the others. Values of Cargo increase earlier than values of the other three predictors (S2 Fig).

### Bayesian clustering

STRUCTURE analysis showed overall a considerable admixture and most of the genetic clusters were shared by different populations. Nevertheless, the populations showed variable degrees of genetic structure, from totally admixed (port 3) to remarkably isolated (port 1) (Fig 4). At both K = 6 and K = 9, the level of admixture of the populations was strongly correlated to the development of the corresponding port (POM regressed against PC1). In other words, mosquitos from busier ports were more genetically admixed and mosquitos from smaller ports were more genetically isolated.

Interestingly, the individuals had mixed ancestry while the populations as a whole were homogeneous (this produced a pattern of "horizontal stripes" in the bar plot) (Fig 4). This could be interpreted as the result of multiple connections between many of these ports for relatively long time. This pattern is different from other studies that analyzed *Ae*. *aegypti*'s population structure at broader spatial scales (several countries and continents); in these studies the ancestry of individual mosquitos was more definite, but mosquitos from the same geographical populations were assigned to different genetic clusters (this produced "vertical stripes" in the STRUCTURE bar plot) [49,50]. A reasonable explanation is that mosquitos migrate more frequently and are more admixed within the same country than between countries and continents; as mosquitos are more distantly related at broader geographical scales, migrant individuals can be more clearly identified.

### Limitations

Main limitation of this study could be the sample size (Table 1), as some of the populations were analyzed with few individuals (especially ports 7 and 11); this could bias the estimation of Fst. Most locations yielded more than 100 individuals, but from few breeding sites. Therefore, we had to compromise between reducing sibling individuals (which could also bias the analysis) and have enough specimens to genotype. After Bonferroni correction 26 pairwise Fst values (24.8% of the total) were not statistically significant and most of them corresponded to the ports with smaller sample size (Table 3). On the other hand, only four (3.8% of the total) remained non-significant if Bonferroni correction was not applied (S1 Dataset). Besides, simulation showed that between 25 and 30 individuals are enough to correctly estimate the allele frequencies with five-nine microsatellite markers [51].

The data from the Philippines Statistics Authority used to elaborate Vessel, Tonnage, Cargo and Passenger was incomplete and it was necessary to estimate the corresponding values for four ports.

However, we believe that these limitations did not undermine the general meaning of our findings.

### Conclusion

Our overall interpretation is that the human ship transportation substantially shapes the dispersal patterns of *Ae*. *aegypti* in the central-western part of the Philippines. Regardless of their geographical location, large ports in highly-populated municipalities exchange individuals of *Ae*. *aegypti* at a faster rate than small-sized ports in less developed municipalities. Insecticide spraying, release of sterile males, release of *Wolbachia* symbionts: whichever vector control strategy will from now on be applied in the Philippines and other archipelagoes, these results should be taken into account.

## Supporting Information

S1 Fig L(K) and ΔK at different values of K. Peaks of ΔK were found at K3, K6, K9. L(K) were averaged over 10 independent replicates.
(PDF)

S2 Fig Scatter plot matrix of the predictors used in univariate and multivariate regressions. Density is non-linearly correlated to predictors related to port size (i.e. Inhabitant and Dock), while Cargo is non-linearly correlated to predictors related to ship connectivity (i.e. Vessel, Tonnage and Passenger). Then, Density and Cargo are totally non-correlated between each other. For detailed description of the predictors refer to Table 2.
(PDF)

S1 Table List and characteristics of the microsatellite markers used in the study. (DOCX)

S2 Table Summary statistics of the predictors used in univariate and multivariate regression. (DOCX)

S3 Table Pearson correlation coefficients between the predictors used in univariate and multivariate regression. (DOCX)

S4 Table Inbreeding coefficients (Fis) for each microsatellite locus across each population. (DOCX)

S5 Table Square roots of variance inflation factors (VIF) of each predictor in two multiple linear regression models. (DOCX)

S1 File Scanned copy of the result of the ethical examination. (PDF)

S1 Dataset Zipped folder containing the dataset organized in separate Excel sheets. (ZIP)

## References

[1] Bhatt S, Gething PW, Brady OJ, Messina JP, Farlow AW, Moyes CL, et al The global distribution and burden of dengue. Nature. Nature Publishing Group, a division of Macmillan Publishers Limited. All Rights Reserved.; 2013;496: 504–7. 10.1038/nature12060 PMC3651993 23563266

[2] Alphey L. Genetic control of mosquitoes. Annu Rev Entomol. Annual Reviews; 2014;59: 205–24. 10.1146/annurev-ento-011613-162002 24160434

[3] Tsuda Y, Takagi M, Wang S, Wang Z, Tang L. Movement of *Aedes aegypti* (Diptera: Culicidae) Released in a Small Isolated Village on Hainan Island, China. J Med Entomol. Entomological Society of America; 2001;38: 93–98. 10.1603/0022-2585-38.1.93 11268697

[4] Bosio CF, Harrington LC, Jones JW, Sithiprasasna R, Norris DE, Scott TW. Genetic structure of *Aedes aegypti* populations in Thailand using mitochondrial DNA. Am J Trop Med Hyg. 2005;72: 434–42. http://www.ncbi.nlm.nih.gov/pubmed/15827282 15827282

[5] Trpis M, Hausermann W. Dispersal and other population parameters of *Aedes aegypti* in an African village and their possible significance in epidemiology of vector-borne diseases. Am J Trop Med Hyg. 1986;35: 1263–79. http://www.ncbi.nlm.nih.gov/pubmed/3789275 3789275 10.4269/ajtmh.1986.35.1263

[6] Guagliardo SA, Barboza JL, Morrison AC, Astete H, Vazquez-Prokopec G, Kitron U. Patterns of Geographic Expansion of *Aedes aegypti* in the Peruvian Amazon. Barrera R, editor. PLoS Negl Trop Dis. Public Library of Science; 2014;8: e3033 10.1371/journal.pntd.0003033 25101786 PMC4125293

[7] Paupy C, Brengues C, Kamgang B, Hervé J-P, Fontenille D, Simard F. Gene flow between domestic and sylvan populations of *Aedes aegypti* (Diptera: Culicidae) in North Cameroon. J Med Entomol. 2008;45: 391–400. http://www.ncbi.nlm.nih.gov/pubmed/18533431 18533431 10.1603/0022-2585(2008)45[391:gfbdas]2.0.co;2

[8] Soper FL. Dynamics of *Aedes aegypti* distribution and density. Seasonal fluctuations in the Americas. Bull World Health Organ. 1967;36: 536–8. http://www.pubmedcentral.nih.gov/articlerender.fcgi?artid=2476418&tool=pmcentrez&rendertype=abstract 5299446 PMC2476418

[9] Chadee DD. *Aedes aegypti* aboard boats at Port-of-Spain, Trinidad, West Indies (1972–82). Mosq News. 1984;44: 1–3. http://bases.bireme.br/cgi-bin/wxislind.exe/iah/online/?IsisScript=iah/iah.xis&src=google&base=MedCarib&lang=p&nextAction=lnk&exprSearch=8802&indexSearch=ID

[10] Suleman M, Arshad M, Khan K. Yellowfever Mosquito (Diptera: Culicidae) Introduced into Landi Kotal, Pakistan, by Tire Importation. J Med Entomol. Entomological Society of America; 1996;33: 689–693. http://www.ingentaconnect.com/content/esa/jme/1996/00000033/00000004/art00028 10.1093/jmedent/33.4.689 8699469

[11] Sukehiro N, Kida N, Umezawa M, Murakami T, Arai N, Jinnai T, et al First Report on Invasion of Yellow Fever Mosquito, *Aedes aegypti*, at Narita International Airport, Japan in August 2012. Jpn J Infect Dis. 2013;66: 189–194. 23698478 10.7883/yoken.66.189

[12] Hardy OJ. Isolation by distance in a continuous population: reconciliation between spatial autocorrelation analysis and population genetics models. Heredity (Edinb). The Genetical Society of Great Britain; 1999;83: 145. 10.1046/j.1365-2540.1999.00558.x 10469202

[13] Patarro T de F, Guirado MM, Ravazzi LM, Bicudo HEM de C. Genetic structure of *Aedes aegypti* populations determined using pairwise comparisons. Genet Mol Res. 2013;12: 3775–87. 10.4238/2013.September.19.9 24085439

[14] Paduan K dosS., Araújo-Júnior JP, Ribolla PEM. Genetic variability in geographical populations of *Aedes aegypti* (Diptera, Culicidae) in Brazil elucidated by molecular markers. Genet Mol Biol. 2006;29: 391–395. http://bases.bireme.br/cgi-bin/wxislind.exe/iah/online/?IsisScript=iah/iah.xis&src=google&base=LILACS&lang=p&nextAction=lnk&exprSearch=432714&indexSearch=ID

[15] Scarpassa VM, Cardoza TB, Cardoso Junior RP. Population genetics and phylogeography of *Aedes aegypti* (Diptera: Culicidae) from Brazil. Am J Trop Med Hyg. 2008/6/11 ed. 2008;78: 895–903. 18541766

[16] Damal K, Murrell EG, Juliano SA, Conn JE, Loew SS. Phylogeography of *Aedes aegypti* (yellow fever mosquito) in South Florida: mtDNA evidence for human-aided dispersal. Am J Trop Med Hyg. 2013;89: 482–8. 10.4269/ajtmh.13-0102 23918216 PMC3771285

[17] Rasheed SB, Boots M, Frantz AC, Butlin RK. Population structure of the mosquito *Aedes aegypti* (Stegomyia aegypti) in Pakistan. Med Vet Entomol. 2013; 10.1111/mve.12001 23662926

[18] Da Costa-Ribeiro MCV, Lourenço-de-Oliveira R, Failloux A-B. Low gene flow of *Aedes aegypti* between dengue-endemic and dengue-free areas in southeastern and southern Brazil. Am J Trop Med Hyg. 2007;77: 303–9. http://www.ncbi.nlm.nih.gov/pubmed/17690403 17690403

[19] Huber K, Loan L Le, Chantha N, Failloux A-B. Human transportation influences *Aedes aegypti* gene flow in Southeast Asia. Acta Trop. 2004;90: 23–9. 14739019 10.1016/j.actatropica.2003.09.012

[20] Gonçalves da Silva A, Cunha ICL, Santos WS, Luz SLB, Ribolla PEM, Abad-Franch F. Gene flow networks among American *Aedes aegypti* populations. Evol Appl. 2012;5: 664–76. 10.1111/j.1752-4571.2012.00244.x 23144654 PMC3492893

[21] Failloux AB, Darius H, Pasteur N. Genetic differentiation of *Aedes aegypti*, the vector of dengue virus in French Polynesia. J Am Mosq Control Assoc. 1995;11: 457–62. http://www.ncbi.nlm.nih.gov/pubmed/8825508 8825508

[22] WPRO. World Health Organization—Western Pacific Region—Health Information and Intelligence Platform (HIIP)—Philippines Indicator Profile by Indicator Group [Internet]. 2013. http://hiip.wpro.who.int/portal/DataAnalytics/ViewPredefinedTables.aspx

[23] Duncombe J, Espino F, Marollano K, Velazco A, Ritchie SA, Hu W-B, et al Characterising the spatial dynamics of sympatric *Aedes aegypti* and Aedes albopictus populations in the Philippines. Geospat Health. 2013;8: 255–65. http://www.ncbi.nlm.nih.gov/pubmed/24258900 24258900 10.4081/gh.2013.71

[24] Edillo FE, Roble ND, Otero ND. The key breeding sites by pupal survey for dengue mosquito vectors, *Aedes aegypti* (Linnaeus) and Aedes albopictus (Skuse), in Guba, Cebu City, Philippines. Southeast Asian J Trop Med Public Health. 2012;43: 1365–74. http://www.ncbi.nlm.nih.gov/pubmed/23413699 23413699

[25] Mahilum MM, Ludwig M, Madon MB, Becker N. Evaluation of the present dengue situation and control strategies against *Aedes aegypti* in Cebu City, Philippines. J Vector Ecol. 2005;30: 277–83. http://www.ncbi.nlm.nih.gov/pubmed/16599163 16599163

[26] Schultz GW. Seasonal abundance of dengue vectors in Manila, Republic of the Philippines. Southeast Asian J Trop Med Public Health. 1993;24: 369–75. http://www.ncbi.nlm.nih.gov/pubmed/8266245 8266245

[27] Higa Y, Bertuso AG, Tokuhisa A, Nagata N, Sawabe K. Studies on the distribution of dengue vectors collected from used tires in Luzon Island, Philippines. The 64th Annual Meeting of the Japanese Society of Medical Entomology and Zoology. 2012 p. 48 (in Japanese).

[28] Sawabe K, Kasai S, Higa Y. Evaluation of insecticide susceptibility of mosquito vectors by genotyping. File No: 2010225032A0003, 201225032A0004 (in Japanese) [Internet]. 2014. https://mhlw-grants.niph.go.jp/niph/search/NIDD00.do?resrchNum=201225032A#selectHokoku

[29] Huang Y-M. Contributions to the Mosquito Fauna of Southeast Asia. XIV. The Subgenus Stegomyia of Aedes in Southeast Asia I—The Scutellaris Group of Species. Contrib Am Entomol Inst. 1972;9: 1–109.

[30] Huang Y-M. Medical Entomology Studies—XI. The Subgenus Stegomyia of Aedes in the Oriental Region with Keys to the Species (Diptera: Culicidae). Contrib Am Entomol Inst. 1979;15: 1–79.

[31] Higa Y, Toma T, Tsuda Y, Miyagi I. A multiplex PCR-based molecular identification of five morphologically related, medically important subgenus Stegomyia mosquitoes from the genus Aedes (Diptera: Culicidae) found in the Ryukyu Archipelago, Japan. Jpn J Infect Dis. 2010;63: 312–6. http://www.ncbi.nlm.nih.gov/pubmed/20858995 20858995

[32] Colton YM, Chadee DD, Severson DW. Natural skip oviposition of the mosquito *Aedes aegypti* indicated by codominant genetic markers. Med Vet Entomol. 2003;17: 195–204. http://www.ncbi.nlm.nih.gov/pubmed/12823837 12823837 10.1046/j.1365-2915.2003.00424.x

[33] Oetting WS, Lee HK, Flanders DJ, Wiesner GL, Sellers TA, King RA. Linkage analysis with multiplexed short tandem repeat polymorphisms using infrared fluorescence and M13 tailed primers. Genomics. 1995;30: 450–8. 8825630 10.1006/geno.1995.1264

[34] Slotman MA, Kelly NB, Harrington LC, Kitthawee S, Jones JW, Scott TW, et al Polymorphic microsatellite markers for studies of *Aedes aegypti* (Diptera: Culicidae), the vector of dengue and yellow fever. Mol Ecol Notes. 2007;7: 168–171.

[35] Chambers EW, Meece JK, McGowan JA, Lovin DD, Hemme RR, Chadee DD, et al Microsatellite isolation and linkage group identification in the yellow fever mosquito *Aedes aegypti* . J Hered. 2007;98: 202–10. 17420178 10.1093/jhered/esm015

[36] Lovin DD, Washington KO, deBruyn B, Hemme RR, Mori A, Epstein SR, et al Genome-based polymorphic microsatellite development and validation in the mosquito *Aedes aegypti* and application to population genetics in Haiti. BMC Genomics. 2009;10: 590 10.1186/1471-2164-10-590 20003193 PMC3087561

[37] Ravel S, Hervé J-P, Diarrassouba S, Kone A, Cuny G, Herve JP. Microsatellite markers for population genetic studies in *Aedes aegypti* (Diptera: Culicidae) from Côte d’Ivoire: evidence for a microgeographic genetic differentiation of mosquitoes from Bouaké. Acta Trop. 2002/3/21 ed. 2002;82: 39–49. 11904102 10.1016/s0001-706x(02)00028-1

[38] Huber K, Mousson L, Rodhain F, Failloux A-B. Isolation and variability of polymorphic microsatellite loci in *Aedes aegypti*, the vector of dengue viruses. Mol Ecol Notes. 2001;1: 219–222.

[39] Raymond M, Rousset F. GENEPOP (Version 1.2): Population Genetics Software for Exact Tests and Ecumenicism. J Hered. 1995;86: 248–249. http://jhered.oxfordjournals.org/content/86/3/248.citation

[40] Rousset F. genepop’007: a complete re-implementation of the genepop software for Windows and Linux. Mol Ecol Resour. 2008;8: 103–6. 10.1111/j.1471-8286.2007.01931.x 21585727

[41] Weir B, Cockerham C. Estimating F-Statistics for the Analysis of Population Structure. Evolution (N Y). 1984;38: 1358–1370. 10.1111/j.1558-5646.1984.tb05657.x 28563791

[42] Excoffier L, Lischer HEL. Arlequin suite ver 3.5: a new series of programs to perform population genetics analyses under Linux and Windows. Mol Ecol Resour. 2010;10: 564–7. 10.1111/j.1755-0998.2010.02847.x 21565059

[43] Van den Berg RA, Hoefsloot HCJ, Westerhuis JA, Smilde AK, van der Werf MJ. Centering, scaling, and transformations: improving the biological information content of metabolomics data. BMC Genomics. 2006;7: 142 16762068 10.1186/1471-2164-7-142 PMC1534033

[44] Jensen JL, Bohonak AJ, Kelley ST. Isolation by distance, web service. BMC Genet. 2005;6: 13 15760479 10.1186/1471-2156-6-13 PMC1079815

[45] Pritchard JK, Stephens M, Donnelly P. Inference of Population Structure Using Multilocus Genotype Data. Genetics. 2000;155: 945–959. http://www.genetics.org/content/155/2/945.full.pdf&embedded=true 10835412 10.1093/genetics/155.2.945 PMC1461096

[46] Evanno G, Regnaut S, Goudet J. Detecting the number of clusters of individuals using the software STRUCTURE: a simulation study. Mol Ecol. 2005;14: 2611–20. 15969739 10.1111/j.1365-294X.2005.02553.x

[47] Hubisz MJ, Falush D, Stephens M, Pritchard JK. Inferring weak population structure with the assistance of sample group information. Mol Ecol Resour. 2009;9: 1322–32. 10.1111/j.1755-0998.2009.02591.x 21564903 PMC3518025

[48] Brown JE, Obas V, Morley V, Powell JR. Phylogeography and spatio-temporal genetic variation of *Aedes aegypti* (Diptera: Culicidae) populations in the Florida Keys. J Med Entomol. 2013;50: 294–9. http://www.ncbi.nlm.nih.gov/pubmed/23540116 23540116 10.1603/me12173 PMC3721520

[49] Monteiro FA, Shama R, Martins AJ, Gloria-Soria A, Brown JE, Powell JR. Genetic Diversity of Brazilian *Aedes aegypti*: Patterns following an Eradication Program. PLoS Negl Trop Dis. Public Library of Science; 2014;8: e3167. 10.1371/journal.pntd.0003167 PMC4169244 25233218

[50] Brown JE, McBride CS, Johnson P, Ritchie S, Paupy C, Bossin H, et al Worldwide patterns of genetic differentiation imply multiple “domestications” of *Aedes aegypti*, a major vector of human diseases. Proc Biol Sci. 2011;278: 2446–54. 10.1098/rspb.2010.2469 21227970 PMC3125627

[51] Hale ML, Burg TM, Steeves TE. Sampling for microsatellite-based population genetic studies: 25 to 30 individuals per population is enough to accurately estimate allele frequencies. PLoS One. 2012;7: e45170 10.1371/journal.pone.0045170 22984627 PMC3440332

