# Supplementary material for: Human-Mediated Marine Dispersal Influences the Population Structure of Aedes aegypti in the Philippine Archipelago
Source: PLoS Negl Trop Dis. 2015 Jun 3;9(6):e0003829. doi: 10.1371/journal.pntd.0003829 (PMC4454683; doi:10.1371/journal.pntd.0003829)
Supplement: S1 Fig — Peaks of ΔK were found at K3, K6, K9. L(K) were averaged over 10 independent replicates. (PDF) [file pntd.0003829.s001.pdf]

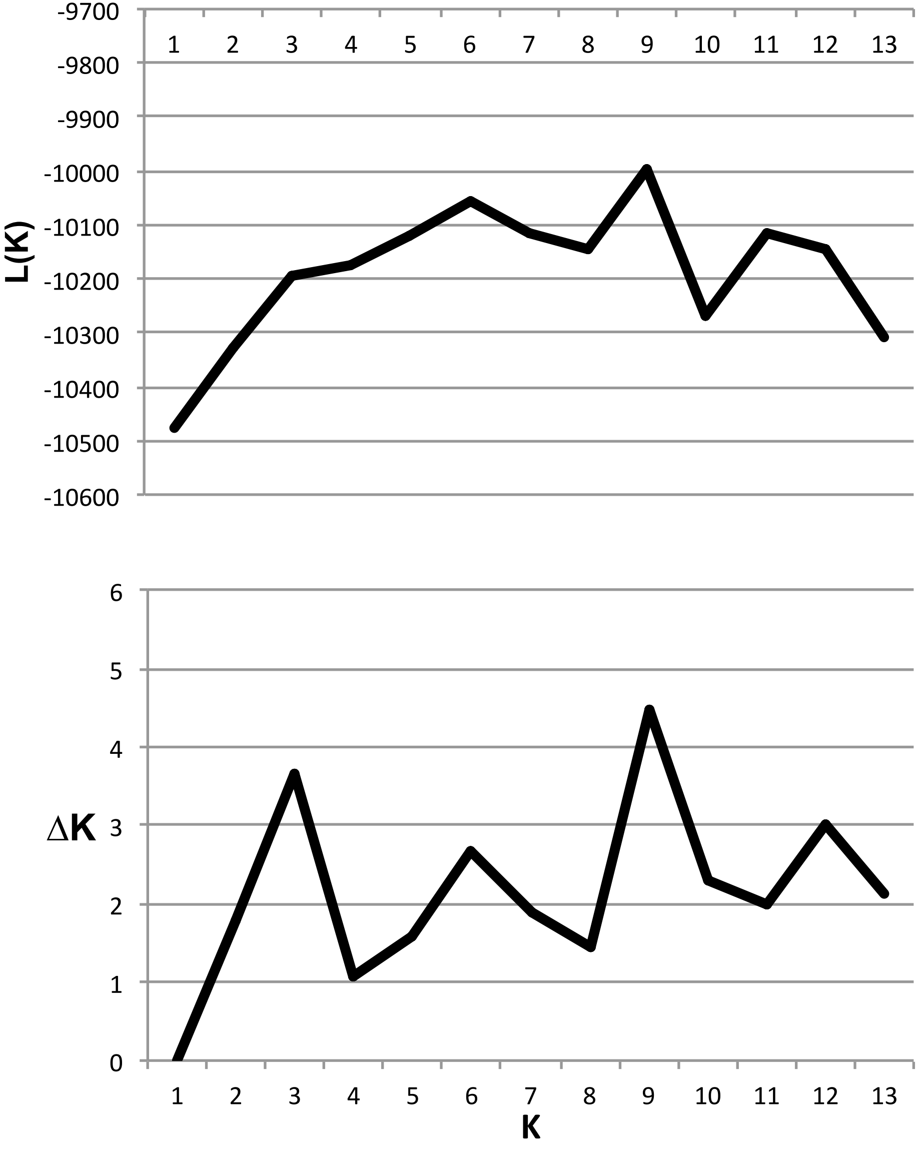

Fig. S1. L(K) and ΔK at different values of K. Peaks of ΔK were found at K3, K6, K9. B. L(K) are averaged over 10 independent replicates.
