# Supplementary material for: Human-Mediated Marine Dispersal Influences the Population Structure of Aedes aegypti in the Philippine Archipelago
Source: PLoS Negl Trop Dis. 2015 Jun 3;9(6):e0003829. doi: 10.1371/journal.pntd.0003829 (PMC4454683; doi:10.1371/journal.pntd.0003829)
Supplement: S2 Fig — Density is non-linearly correlated to predictors related to port size (i.e. Inhabitant and Dock), while Cargo is non-linearly correlated to predictors related to ship connectivity (i.e. Vessel, Tonnage and Passenger). Then, Density and Cargo are totally non-correlated between each other. For detailed description of the predictors refer to Table 2. (PDF) [file pntd.0003829.s002.pdf]

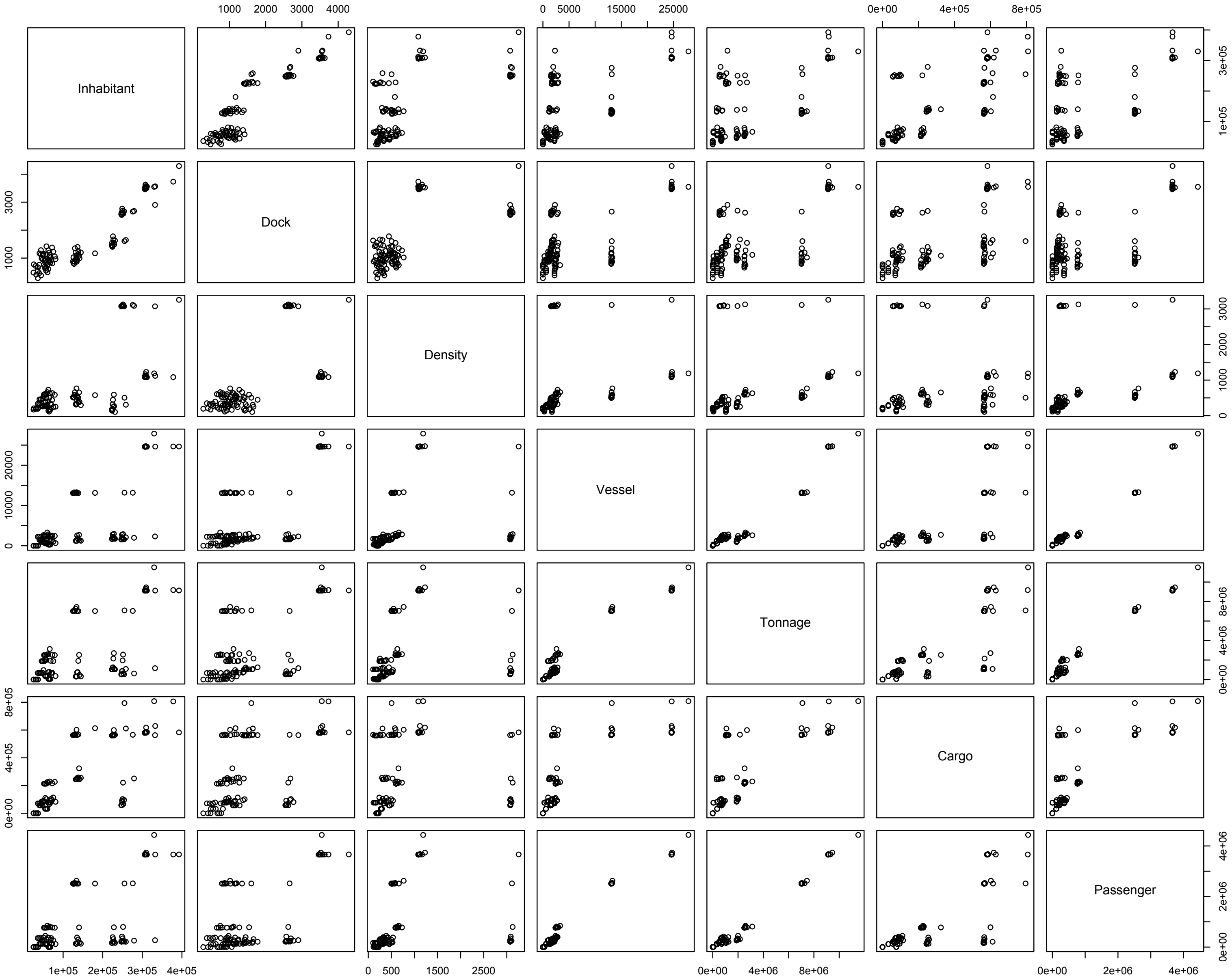

S2 Fig. Scatter plot matrix of the predictors used in univariate and multivariate regressions. Density is non-linearly correlated to predictors related to port size (i.e. Inhabitant and Dock), while Cargo is non-linearly correlated to predictors related to ship connectivity (i.e. Vessel, Tonnage and Passenger). Then, Density and Cargo are totally non-correlated between each other. For detailed description of the predictors refer to Table 2.
