# Supplementary material for: Human-Mediated Marine Dispersal Influences the Population Structure of Aedes aegypti in the Philippine Archipelago
Source: PLoS Negl Trop Dis. 2015 Jun 3;9(6):e0003829. doi: 10.1371/journal.pntd.0003829 (PMC4454683; doi:10.1371/journal.pntd.0003829)
Supplement: S1 Table — (DOCX) [file pntd.0003829.s003.docx]

**Table S1.** **List and characteristics of the microsatellite markers used in the study.**

| **Marker** | **Forward Primer*** | **Reverse Primer** | **T_a_ (°C)** | **Repeat Unit** | **Alleles** | **Source** |
| --- | --- | --- | --- | --- | --- | --- |
| H08 | AAAAACCACGATCACCGAAG | ACGCGATCACACACTGAAAATG | 63 | 3 bp | 4 | Chambers et al. |
| AG3 | CGCCAAAACTGAAAACTGAA | AAGGGCGGTGATGACTTTCT | 67 | 2 bp | 6 | Slotman et al. |
| 69TGA1 | CACCTCCGCTAGAGAACTGG | CGAATAGGGCAATCCTGAAA | 62 | 3 bp | 6 | Lovin et al. |
| CT2 | CGCAGTAGGCGATATTCGTT | ACCACCACCAACACCATTCT | 64 | 2 bp | 5 | Slotman et al. |
| AG5 | TGATCTTGAGAAGGCATCCA | CGTTATCCTTTCATCACTTGTTTG | 59 | 2 bp | 11 | Slotman et al. |
| AC2 | AATACAACGCGATCGACTCC | AACGATTAGCTGCTCCGAAA | 63 | 2 bp | 4 | Slotman et al. |
| 12ATG1 | TCTGCGCAACACTAAGCATC | AAAAAGCACACCGTTCTCGT | 62 | 3 bp | 5 | Lovin et al. |
| **Labelled Primer**** | | | | | | |
| M13 | GTAAAACGACGGCCAG | NA | NA | NA | NA | NA |

* An M13-tail with the same sequence as the M13-labelled primer was added on the 5'-end of each of the forward primers.

** The 5'-end of the M13 primer was labelled with FAM (see methods).

T_a_ = T of annealing
