# Supplementary material for: Human-Mediated Marine Dispersal Influences the Population Structure of Aedes aegypti in the Philippine Archipelago
Source: PLoS Negl Trop Dis. 2015 Jun 3;9(6):e0003829. doi: 10.1371/journal.pntd.0003829 (PMC4454683; doi:10.1371/journal.pntd.0003829)
Supplement: S2 Table — (DOCX) [file pntd.0003829.s004.docx]

**S2 Table.** **Summary statistics of the predictors used in univariate and multivariate regression.**

| **Predictor** | **Average** | **Median** | **St. deviation** | **Minimum** | **Maximum** |
| --- | --- | --- | --- | --- | --- |
| Distance | 223.8 | 139 | 208.4 | 15 | 702 |
| Inhabitant | 186053 | 157557 | 123491 | 34782 | 551999 |
| Density | 994.6 | 602 | 1004 | 141 | 4152 |
| Dock | 1975 | 1555 | 1190 | 353 | 6002 |
| Vessel | 7628 | 3496 | 8630 | 867 | 37686 |
| Tonnage | 3265056 | 1569410 | 3618420 | 107084 | 16134280 |
| Cargo | 385203 | 284468 | 269593 | 65129 | 1142842 |
| Passenger | 1127635 | 385498 | 1406384 | 24961 | 6167947 |

N=105. Refer to Table 2 for detailed description of the predictors.
