# Supplementary material for: Human-Mediated Marine Dispersal Influences the Population Structure of Aedes aegypti in the Philippine Archipelago
Source: PLoS Negl Trop Dis. 2015 Jun 3;9(6):e0003829. doi: 10.1371/journal.pntd.0003829 (PMC4454683; doi:10.1371/journal.pntd.0003829)
Supplement: S3 Table — (DOCX) [file pntd.0003829.s005.docx]

**S3 Table.** **Pearson correlation coefficients between the predictors used in univariate and multivariate regression.**

|  | **Distance** | **Inhabitant** | **Density** | **Dock** | **Vessel** | **Tonnage** | **Cargo** |
| --- | --- | --- | --- | --- | --- | --- | --- |
| **Distance** |  |  |  |  |  |  |  |
| **Inhabitant** | 0.2 |  |  |  |  |  |  |
| **Density** | -0.15 | 0.61 |  |  |  |  |  |
| **Dock** | 0.09 | 0.9 | 0.68 |  |  |  |  |
| **Vessel** | -0.11 | 0.61 | 0.2 | 0.67 |  |  |  |
| **Tonnage** | -0.11 | 0.58 | 0.19 | 0.6 | 0.95 |  |  |
| **Cargo** | 0.18 | 0.69 | 0.01 | 0.47 | 0.71 | 0.77 |  |
| **Passenger** | -0.12 | 0.59 | 0.22 | 0.62 | 0.98 | 0.99 | 0.75 |

N=105. Refer to Table 2 for detailed description of each predictor.
