# Supplementary material for: Human-Mediated Marine Dispersal Influences the Population Structure of Aedes aegypti in the Philippine Archipelago
Source: PLoS Negl Trop Dis. 2015 Jun 3;9(6):e0003829. doi: 10.1371/journal.pntd.0003829 (PMC4454683; doi:10.1371/journal.pntd.0003829)
Supplement: S4 Table — (DOCX) [file pntd.0003829.s006.docx]

**S4 Table. Inbreeding coefficients (Fis) for each microsatellite locus across each population.**

| **Population** | **CT2** | **AG5** | **AC2** | **H08** | **AG3** | **69TGA1** | **12ATG1** |
| --- | --- | --- | --- | --- | --- | --- | --- |
| **1** | -0.06 | **0.06** | -0.05 | 0.28 | -0.03 | -0.08 | 0.18 |
| **2** | -0.15 | -0.20 | -0.05 | -0.16 | 0.20 | -0.03 | -0.05 |
| **3** | 0.05 | 0.13 | 0.19 | -0.01 | -0.02 | -0.03 | 0.08 |
| **4** | 0.14 | 0.08 | 0.26 | 0.00 | -0.08 | -0.11 | 0.09 |
| **5** | -0.06 | 0.03 | 0.22 | -0.07 | 0.05 | 0.04 | **0.32** |
| **6** | 0.14 | 0.07 | 0.06 | -0.18 | -0.22 | **0.22** | 0.11 |
| **7** | 0.03 | -0.22 | 0.07 | 0.36 | -0.08 | 0.00 | 0.10 |
| **8** | -0.10 | 0.05 | 0.04 | -0.12 | 0.00 | -0.13 | 0.03 |
| **9** | -0.21 | 0.07 | 0.03 | 0.28 | 0.14 | **-0.31** | 0.18 |
| **10** | 0.27 | **0.25** | -0.02 | **0.34** | 0.01 | **0.40** | 0.04 |
| **11** | 0.39 | 0.00 | 0.14 | 0.34 | 0.27 | -0.03 | 0.14 |
| **12** | -0.14 | 0.11 | 0.25 | 0.06 | 0.10 | -0.01 | -0.04 |
| **13** | 0.18 | 0.13 | 0.05 | 0.30 | -0.09 | -0.03 | 0.02 |
| **14** | 0.02 | 0.01 | 0.23 | -0.20 | -0.11 | 0.33 | 0.13 |
| **15** | -0.02 | **0.28** | **0.40** | 0.00 | 0.12 | -0.01 | -0.05 |

Bold shows significant departure from HWE after Bonferroni sequential correction.
