# Supplementary material for: Human-Mediated Marine Dispersal Influences the Population Structure of Aedes aegypti in the Philippine Archipelago
Source: PLoS Negl Trop Dis. 2015 Jun 3;9(6):e0003829. doi: 10.1371/journal.pntd.0003829 (PMC4454683; doi:10.1371/journal.pntd.0003829)
Supplement: S5 Table — (DOCX) [file pntd.0003829.s007.docx]

**S5 Table.** **Square roots of variance inflation factors (VIF) of each predictor in two multiple linear regression models.**

|  | **Sqrt(VIF)** | |
| --- | --- | --- |
| **Predictor** | **Linear model A^1^** | **Linear model B^2^** |
| **Distance** | 1.29 | 1.23 |
| **Inhabitant** | 7.03 | 5.01 |
| **Density** | 2.58 | 2.02 |
| **Dock** | 6.22 | 4.17 |
| **Vessel** | 9.35 | 2.49 |
| **Tonnage** | 13.23 | - |
| **Cargo** | 4.96 | 3.2 |
| **Passenger** | 18.48 | - |

^1^A multiple linear model run with all the predictors. ^2^A multiple linear model run with six predictors. Refer to Table 2 for detailed description of each predictor.
