# Supplementary material for: Human-Mediated Marine Dispersal Influences the Population Structure of Aedes aegypti in the Philippine Archipelago
Source: PLoS Negl Trop Dis. 2015 Jun 3;9(6):e0003829. doi: 10.1371/journal.pntd.0003829 (PMC4454683; doi:10.1371/journal.pntd.0003829)
Supplement: S1 File — (PDF) [file pntd.0003829.s008.pdf]

Letter of Notice on the Examination Result by Ethical Committee

Date June 12, 2013

Kouichi Morita
Dean
Institute of Tropical Medicine
Nagasaki University

Applicant: Yukiko Higa
Approval No. 130606105
Title of Investigation: Distribution of dengue vector infestation, pyrethroid-related insecticide resistance and population genetics of the vectors in the Philippines.

The protocol and execution plan for the above-entitled investigation has been reviewed and considered appropriate without any ethical problems by the Ethical Committee of Institute of Tropical Medicine, Nagasaki University held on June 6, 2013.

Memorandum

Decision to the submission	☐ Approved<br>☐ Conditionally Approved<br>☐ Recommendation for Revision of Protocol<br>☐ Rejected<br>■ Not Pertinence
Reason for the decision	This study focuses on mosquito collection only and therefore has no ethical problem. Since sampling from human such as blood and personal information is not involved, the protocol and execution plan are of non-pertinence for the committee.
