# Supplementary material for: Human-Mediated Marine Dispersal Influences the Population Structure of Aedes aegypti in the Philippine Archipelago
Source: PLoS Negl Trop Dis. 2015 Jun 3;9(6):e0003829. doi: 10.1371/journal.pntd.0003829 (PMC4454683; doi:10.1371/journal.pntd.0003829)
Supplement: S1 Dataset — (ZIP) [file pntd.0003829.s009.zip › S1_Dataset/Arlequin_results.htm]

```
Arlequin results


////////////////////////////////////////////////////////////////////
RUN NUMBER 1 (15/01/15 at 12:14:22)
////////////////////////////////////////////////////////////////////


Project information:
--------------------
	NbSamples      =  15
	DataType       =  STANDARD
	GenotypicData  =  1
	GameticPhase   =  0
	RecessiveData  =  0

==============================
Settings used for Calculations
==============================

 General settings:
 -----------------
        Deletion Weight                = 1
        Transition Weight Weight       = 1
        Tranversion Weight Weight      = 1
        Epsilon Value                  = 1e-07
        Significant digits for output  = 5
        Infer haplotype definition from distance matrix
        Alllowed level of missing data = 0.05

 Active Tasks:
 -------------

    Standard indices:
    -----------------

    Molecular Diversity:
    --------------------
        Molecular Distance :Pairwise differences
        GammaA Value       = 0
        Theta estimators   : 

    Population pairwise Fst values:
    -------------------------------
        Compute pairwise differences
        Compute relative population sizes
        Compute coancestry coefficients
        Compute Slatkin's distances
        No. of permutations for significance = 10000
        No. of permutations for Mantel test  = 1000

       Distance matrix:
            Compute distance matrix
            Molecular distance : Pairwise differences
            Gamma a value      = 0


==============================================================================
== ANALYSES AT THE INTRA-POPULATION LEVEL
==============================================================================


===============================================================================
== Sample : 	Ambulong
===============================================================================


===============================
== Standard diversity indices : (Ambulong)
===============================

Reference: Nei, M., 1987.
No. of gene copies       : 132
No. of loci              : 7
No. of usable loci       : 7 loci with less than  5.00 % missing data
No. of polymorphic loci  : 7


Results are only shown for polymorphic loci
-------------------------------------------------
           Num.
           gene     Num.        Obs.      Exp.
Locus#    copies   alleles      Het.       Het
-------------------------------------------------
     1       132         3   0.51515   0.48531
     2       132         5   0.60606   0.64157
     3       132         4   0.59091   0.56269
     4       132         4   0.31818   0.44067
     5       132         4   0.71212   0.68980
     6       132         3   0.60606   0.56061
     7       132         4   0.50000   0.61080
-------------------------------------------------
  Mean   132.000     3.857   0.54978   0.57021
  s.d.     0.000     0.690   0.12362   0.08678
-------------------------------------------------


================================
== Molecular diversity indices : (Ambulong)
================================

           Tajima, F., 1983.
           Tajima, F. 1993.
           Nei, M., 1987.
           Zouros, E., 1979.
           Ewens, W.J. 1972.
Sample size                                  : 132.00000
No. of haplotypes                            : 93

Allowed level of missing data                : 5.00000 %
Number of polymorphic loci                   : 7
Number of usable loci                        : 7


Average gene diversity over loci              :     0.570206 +/-     0.317542

(Standard deviations are for both sampling and stochastic processes)


===============================================================================
== Sample : 	Balanacan
===============================================================================


===============================
== Standard diversity indices : (Balanacan)
===============================

Reference: Nei, M., 1987.
No. of gene copies       : 48
No. of loci              : 7
No. of usable loci       : 7 loci with less than  5.00 % missing data
No. of polymorphic loci  : 7


Results are only shown for polymorphic loci
-------------------------------------------------
           Num.
           gene     Num.        Obs.      Exp.
Locus#    copies   alleles      Het.       Het
-------------------------------------------------
     1        48         4   0.62500   0.54433
     2        48         5   0.87500   0.73316
     3        48         3   0.66667   0.63741
     4        48         3   0.62500   0.54167
     5        48         5   0.62500   0.78014
     6        48         3   0.62500   0.60638
     7        48         4   0.75000   0.71454
-------------------------------------------------
  Mean    48.000     3.857   0.68452   0.65109
  s.d.     0.000     0.900   0.09579   0.09396
-------------------------------------------------


================================
== Molecular diversity indices : (Balanacan)
================================

           Tajima, F., 1983.
           Tajima, F. 1993.
           Nei, M., 1987.
           Zouros, E., 1979.
           Ewens, W.J. 1972.
Sample size                                  : 48.00000
No. of haplotypes                            : 44

Allowed level of missing data                : 5.00000 %
Number of polymorphic loci                   : 7
Number of usable loci                        : 7


Average gene diversity over loci              :     0.651089 +/-     0.360941

(Standard deviations are for both sampling and stochastic processes)


===============================================================================
== Sample : 	Batangas
===============================================================================


===============================
== Standard diversity indices : (Batangas)
===============================

Reference: Nei, M., 1987.
No. of gene copies       : 112
No. of loci              : 7
No. of usable loci       : 6 loci with less than  5.00 % missing data
No. of polymorphic loci  : 6


Results are only shown for polymorphic loci
-------------------------------------------------
           Num.
           gene     Num.        Obs.      Exp.
Locus#    copies   alleles      Het.       Het
-------------------------------------------------
     1       112         3   0.37500   0.39591
     2       112         7   0.62500   0.71718
     3       112         4   0.53571   0.66120
     4       112         3   0.48214   0.47539
     5       102         5   0.70588   0.68919
     6       112         4   0.66071   0.64398
     7       112         4   0.55357   0.60167
-------------------------------------------------
  Mean   110.571     4.286   0.56258   0.59779
  s.d.     3.780     1.380   0.11293   0.11868
-------------------------------------------------


================================
== Molecular diversity indices : (Batangas)
================================

           Tajima, F., 1983.
           Tajima, F. 1993.
           Nei, M., 1987.
           Zouros, E., 1979.
           Ewens, W.J. 1972.
Sample size                                  : 112.00000
No. of haplotypes                            : 100

Allowed level of missing data                : 5.00000 %
Number of polymorphic loci                   : 6
Number of usable loci                        : 6


Average gene diversity over loci              :     0.582556 +/-     0.331080

(Standard deviations are for both sampling and stochastic processes)


===============================================================================
== Sample : 	Brookes_Point
===============================================================================


===============================
== Standard diversity indices : (Brookes_Point)
===============================

Reference: Nei, M., 1987.
No. of gene copies       : 98
No. of loci              : 7
No. of usable loci       : 7 loci with less than  5.00 % missing data
No. of polymorphic loci  : 7


Results are only shown for polymorphic loci
-------------------------------------------------
           Num.
           gene     Num.        Obs.      Exp.
Locus#    copies   alleles      Het.       Het
-------------------------------------------------
     1        98         2   0.34694   0.40332
     2        98         6   0.73469   0.79508
     3        98         3   0.42857   0.57963
     4        98         3   0.51020   0.51147
     5        98         5   0.79592   0.73974
     6        98         4   0.75510   0.67894
     7        98         4   0.55102   0.60656
-------------------------------------------------
  Mean    98.000     3.857   0.58892   0.61639
  s.d.     0.000     1.345   0.17494   0.13473
-------------------------------------------------


================================
== Molecular diversity indices : (Brookes_Point)
================================

           Tajima, F., 1983.
           Tajima, F. 1993.
           Nei, M., 1987.
           Zouros, E., 1979.
           Ewens, W.J. 1972.
Sample size                                  : 98.00000
No. of haplotypes                            : 83

Allowed level of missing data                : 5.00000 %
Number of polymorphic loci                   : 7
Number of usable loci                        : 7


Average gene diversity over loci              :     0.616393 +/-     0.340578

(Standard deviations are for both sampling and stochastic processes)


===============================================================================
== Sample : 	Cajidiocan
===============================================================================


===============================
== Standard diversity indices : (Cajidiocan)
===============================

Reference: Nei, M., 1987.
No. of gene copies       : 106
No. of loci              : 7
No. of usable loci       : 7 loci with less than  5.00 % missing data
No. of polymorphic loci  : 7


Results are only shown for polymorphic loci
-------------------------------------------------
           Num.
           gene     Num.        Obs.      Exp.
Locus#    copies   alleles      Het.       Het
-------------------------------------------------
     1       106         4   0.64151   0.60323
     2       106         6   0.67925   0.70261
     3       106         4   0.50943   0.64888
     4       106         2   0.37736   0.35364
     5       106         5   0.62264   0.65211
     6       106         5   0.58491   0.60773
     7       106         4   0.49057   0.71716
-------------------------------------------------
  Mean   106.000     4.286   0.55795   0.61219
  s.d.     0.000     1.254   0.10497   0.12185
-------------------------------------------------


================================
== Molecular diversity indices : (Cajidiocan)
================================

           Tajima, F., 1983.
           Tajima, F. 1993.
           Nei, M., 1987.
           Zouros, E., 1979.
           Ewens, W.J. 1972.
Sample size                                  : 106.00000
No. of haplotypes                            : 92

Allowed level of missing data                : 5.00000 %
Number of polymorphic loci                   : 7
Number of usable loci                        : 7


Average gene diversity over loci              :     0.612194 +/-     0.338309

(Standard deviations are for both sampling and stochastic processes)


===============================================================================
== Sample : 	Calapan
===============================================================================


===============================
== Standard diversity indices : (Calapan)
===============================

Reference: Nei, M., 1987.
No. of gene copies       : 96
No. of loci              : 7
No. of usable loci       : 7 loci with less than  5.00 % missing data
No. of polymorphic loci  : 7


Results are only shown for polymorphic loci
-------------------------------------------------
           Num.
           gene     Num.        Obs.      Exp.
Locus#    copies   alleles      Het.       Het
-------------------------------------------------
     1        96         3   0.43750   0.50899
     2        94         7   0.63830   0.68520
     3        96         3   0.60417   0.64211
     4        96         3   0.60417   0.51513
     5        96         4   0.77083   0.63421
     6        96         3   0.47917   0.61294
     7        96         4   0.60417   0.68004
-------------------------------------------------
  Mean    95.714     3.857   0.59119   0.61123
  s.d.     0.756     1.464   0.10891   0.07232
-------------------------------------------------


================================
== Molecular diversity indices : (Calapan)
================================

           Tajima, F., 1983.
           Tajima, F. 1993.
           Nei, M., 1987.
           Zouros, E., 1979.
           Ewens, W.J. 1972.
Sample size                                  : 96.00000
No. of haplotypes                            : 83

Allowed level of missing data                : 5.00000 %
Number of polymorphic loci                   : 7
Number of usable loci                        : 7


Average gene diversity over loci              :     0.607174 +/-     0.336210

(Standard deviations are for both sampling and stochastic processes)


===============================================================================
== Sample : 	Cawit
===============================================================================


===============================
== Standard diversity indices : (Cawit)
===============================

Reference: Nei, M., 1987.
No. of gene copies       : 28
No. of loci              : 7
No. of usable loci       : 7 loci with less than  5.00 % missing data
No. of polymorphic loci  : 7


Results are only shown for polymorphic loci
-------------------------------------------------
           Num.
           gene     Num.        Obs.      Exp.
Locus#    copies   alleles      Het.       Het
-------------------------------------------------
     1        28         2   0.50000   0.51587
     2        28         5   0.92857   0.76455
     3        28         3   0.64286   0.68783
     4        28         3   0.28571   0.44180
     5        28         5   0.78571   0.72751
     6        28         3   0.57143   0.57407
     7        28         4   0.57143   0.63228
-------------------------------------------------
  Mean    28.000     3.571   0.61224   0.62056
  s.d.     0.000     1.134   0.20561   0.11684
-------------------------------------------------


================================
== Molecular diversity indices : (Cawit)
================================

           Tajima, F., 1983.
           Tajima, F. 1993.
           Nei, M., 1987.
           Zouros, E., 1979.
           Ewens, W.J. 1972.
Sample size                                  : 28.00000
No. of haplotypes                            : 26

Allowed level of missing data                : 5.00000 %
Number of polymorphic loci                   : 7
Number of usable loci                        : 7


Average gene diversity over loci              :     0.620559 +/-     0.351236

(Standard deviations are for both sampling and stochastic processes)


===============================================================================
== Sample : 	Lucena
===============================================================================


===============================
== Standard diversity indices : (Lucena)
===============================

Reference: Nei, M., 1987.
No. of gene copies       : 112
No. of loci              : 7
No. of usable loci       : 7 loci with less than  5.00 % missing data
No. of polymorphic loci  : 7


Results are only shown for polymorphic loci
-------------------------------------------------
           Num.
           gene     Num.        Obs.      Exp.
Locus#    copies   alleles      Het.       Het
-------------------------------------------------
     1       112         3   0.46429   0.42149
     2       112         7   0.69643   0.73472
     3       112         4   0.64286   0.67165
     4       112         3   0.51786   0.46412
     5       112         5   0.60714   0.60827
     6       112         4   0.67857   0.60135
     7       112         4   0.66071   0.68034
-------------------------------------------------
  Mean   112.000     4.286   0.60969   0.59742
  s.d.     0.000     1.380   0.08713   0.11553
-------------------------------------------------


================================
== Molecular diversity indices : (Lucena)
================================

           Tajima, F., 1983.
           Tajima, F. 1993.
           Nei, M., 1987.
           Zouros, E., 1979.
           Ewens, W.J. 1972.
Sample size                                  : 112.00000
No. of haplotypes                            : 99

Allowed level of missing data                : 5.00000 %
Number of polymorphic loci                   : 7
Number of usable loci                        : 7


Average gene diversity over loci              :     0.597421 +/-     0.331045

(Standard deviations are for both sampling and stochastic processes)


===============================================================================
== Sample : 	Odiongan
===============================================================================


===============================
== Standard diversity indices : (Odiongan)
===============================

Reference: Nei, M., 1987.
No. of gene copies       : 80
No. of loci              : 7
No. of usable loci       : 7 loci with less than  5.00 % missing data
No. of polymorphic loci  : 7


Results are only shown for polymorphic loci
-------------------------------------------------
           Num.
           gene     Num.        Obs.      Exp.
Locus#    copies   alleles      Het.       Het
-------------------------------------------------
     1        80         3   0.47500   0.39462
     2        80         6   0.70000   0.74937
     3        80         3   0.57500   0.59462
     4        80         3   0.37500   0.51930
     5        80         6   0.55000   0.63829
     6        80         3   0.87500   0.67184
     7        80         4   0.50000   0.60570
-------------------------------------------------
  Mean    80.000     4.000   0.57857   0.59625
  s.d.     0.000     1.414   0.16421   0.11372
-------------------------------------------------


================================
== Molecular diversity indices : (Odiongan)
================================

           Tajima, F., 1983.
           Tajima, F. 1993.
           Nei, M., 1987.
           Zouros, E., 1979.
           Ewens, W.J. 1972.
Sample size                                  : 80.00000
No. of haplotypes                            : 57

Allowed level of missing data                : 5.00000 %
Number of polymorphic loci                   : 7
Number of usable loci                        : 7


Average gene diversity over loci              :     0.596248 +/-     0.331609

(Standard deviations are for both sampling and stochastic processes)


===============================================================================
== Sample : 	Puerto_Princesa
===============================================================================


===============================
== Standard diversity indices : (Puerto_Princesa)
===============================

Reference: Nei, M., 1987.
No. of gene copies       : 86
No. of loci              : 7
No. of usable loci       : 7 loci with less than  5.00 % missing data
No. of polymorphic loci  : 7


Results are only shown for polymorphic loci
-------------------------------------------------
           Num.
           gene     Num.        Obs.      Exp.
Locus#    copies   alleles      Het.       Het
-------------------------------------------------
     1        86         4   0.25581   0.35130
     2        86         7   0.53488   0.70889
     3        86         4   0.67442   0.66293
     4        86         3   0.27907   0.42107
     5        86         5   0.72093   0.72558
     6        86         3   0.39535   0.65554
     7        86         5   0.69767   0.72640
-------------------------------------------------
  Mean    86.000     4.429   0.50831   0.60739
  s.d.     0.000     1.397   0.19934   0.15499
-------------------------------------------------


================================
== Molecular diversity indices : (Puerto_Princesa)
================================

           Tajima, F., 1983.
           Tajima, F. 1993.
           Nei, M., 1987.
           Zouros, E., 1979.
           Ewens, W.J. 1972.
Sample size                                  : 86.00000
No. of haplotypes                            : 76

Allowed level of missing data                : 5.00000 %
Number of polymorphic loci                   : 7
Number of usable loci                        : 7


Average gene diversity over loci              :     0.607387 +/-     0.336702

(Standard deviations are for both sampling and stochastic processes)


===============================================================================
== Sample : 	Romblon
===============================================================================


===============================
== Standard diversity indices : (Romblon)
===============================

Reference: Nei, M., 1987.
No. of gene copies       : 38
No. of loci              : 7
No. of usable loci       : 7 loci with less than  5.00 % missing data
No. of polymorphic loci  : 7


Results are only shown for polymorphic loci
-------------------------------------------------
           Num.
           gene     Num.        Obs.      Exp.
Locus#    copies   alleles      Het.       Het
-------------------------------------------------
     1        38         3   0.26316   0.42817
     2        38         9   0.84211   0.84211
     3        38         3   0.47368   0.54623
     4        38         3   0.36842   0.55619
     5        38         5   0.52632   0.71266
     6        38         3   0.57895   0.56188
     7        38         4   0.63158   0.73400
-------------------------------------------------
  Mean    38.000     4.286   0.52632   0.62589
  s.d.     0.000     2.215   0.18732   0.14163
-------------------------------------------------


================================
== Molecular diversity indices : (Romblon)
================================

           Tajima, F., 1983.
           Tajima, F. 1993.
           Nei, M., 1987.
           Zouros, E., 1979.
           Ewens, W.J. 1972.
Sample size                                  : 38.00000
No. of haplotypes                            : 35

Allowed level of missing data                : 5.00000 %
Number of polymorphic loci                   : 7
Number of usable loci                        : 7


Average gene diversity over loci              :     0.625889 +/-     0.350576

(Standard deviations are for both sampling and stochastic processes)


===============================================================================
== Sample : 	Roxas
===============================================================================


===============================
== Standard diversity indices : (Roxas)
===============================

Reference: Nei, M., 1987.
No. of gene copies       : 60
No. of loci              : 7
No. of usable loci       : 7 loci with less than  5.00 % missing data
No. of polymorphic loci  : 7


Results are only shown for polymorphic loci
-------------------------------------------------
           Num.
           gene     Num.        Obs.      Exp.
Locus#    copies   alleles      Het.       Het
-------------------------------------------------
     1        60         3   0.53333   0.46723
     2        60         7   0.70000   0.78701
     3        60         3   0.46667   0.62316
     4        60         2   0.40000   0.42712
     5        60         6   0.70000   0.77910
     6        60         4   0.56667   0.56328
     7        60         4   0.76667   0.73559
-------------------------------------------------
  Mean    60.000     4.143   0.59048   0.62607
  s.d.     0.000     1.773   0.13569   0.14724
-------------------------------------------------


================================
== Molecular diversity indices : (Roxas)
================================

           Tajima, F., 1983.
           Tajima, F. 1993.
           Nei, M., 1987.
           Zouros, E., 1979.
           Ewens, W.J. 1972.
Sample size                                  : 60.00000
No. of haplotypes                            : 54

Allowed level of missing data                : 5.00000 %
Number of polymorphic loci                   : 7
Number of usable loci                        : 7


Average gene diversity over loci              :     0.626069 +/-     0.347381

(Standard deviations are for both sampling and stochastic processes)


===============================================================================
== Sample : 	San_Agustin
===============================================================================


===============================
== Standard diversity indices : (San_Agustin)
===============================

Reference: Nei, M., 1987.
No. of gene copies       : 128
No. of loci              : 7
No. of usable loci       : 7 loci with less than  5.00 % missing data
No. of polymorphic loci  : 7


Results are only shown for polymorphic loci
-------------------------------------------------
           Num.
           gene     Num.        Obs.      Exp.
Locus#    copies   alleles      Het.       Het
-------------------------------------------------
     1       128         3   0.10938   0.13349
     2       122         9   0.68852   0.78892
     3       128         3   0.56250   0.58994
     4       128         2   0.18750   0.26575
     5       128         4   0.68750   0.62857
     6       128         3   0.60938   0.59412
     7       128         4   0.71875   0.73142
-------------------------------------------------
  Mean   127.143     4.000   0.50907   0.53317
  s.d.     2.268     2.309   0.25301   0.24221
-------------------------------------------------


================================
== Molecular diversity indices : (San_Agustin)
================================

           Tajima, F., 1983.
           Tajima, F. 1993.
           Nei, M., 1987.
           Zouros, E., 1979.
           Ewens, W.J. 1972.
Sample size                                  : 128.00000
No. of haplotypes                            : 104

Allowed level of missing data                : 5.00000 %
Number of polymorphic loci                   : 7
Number of usable loci                        : 7


Average gene diversity over loci              :     0.522814 +/-     0.294802

(Standard deviations are for both sampling and stochastic processes)


===============================================================================
== Sample : 	San_Jose
===============================================================================


===============================
== Standard diversity indices : (San_Jose)
===============================

Reference: Nei, M., 1987.
No. of gene copies       : 62
No. of loci              : 7
No. of usable loci       : 7 loci with less than  5.00 % missing data
No. of polymorphic loci  : 7


Results are only shown for polymorphic loci
-------------------------------------------------
           Num.
           gene     Num.        Obs.      Exp.
Locus#    copies   alleles      Het.       Het
-------------------------------------------------
     1        62         3   0.54839   0.55896
     2        62         5   0.61290   0.62031
     3        62         3   0.48387   0.62454
     4        60         3   0.56667   0.47401
     5        62         5   0.67742   0.61132
     6        62         3   0.45161   0.66896
     7        62         4   0.61290   0.70492
-------------------------------------------------
  Mean    61.714     3.714   0.56482   0.60900
  s.d.     0.756     0.951   0.07847   0.07516
-------------------------------------------------


================================
== Molecular diversity indices : (San_Jose)
================================

           Tajima, F., 1983.
           Tajima, F. 1993.
           Nei, M., 1987.
           Zouros, E., 1979.
           Ewens, W.J. 1972.
Sample size                                  : 62.00000
No. of haplotypes                            : 56

Allowed level of missing data                : 5.00000 %
Number of polymorphic loci                   : 7
Number of usable loci                        : 7


Average gene diversity over loci              :     0.604669 +/-     0.336845

(Standard deviations are for both sampling and stochastic processes)


===============================================================================
== Sample : 	Torrijos
===============================================================================


===============================
== Standard diversity indices : (Torrijos)
===============================

Reference: Nei, M., 1987.
No. of gene copies       : 96
No. of loci              : 7
No. of usable loci       : 7 loci with less than  5.00 % missing data
No. of polymorphic loci  : 7


Results are only shown for polymorphic loci
-------------------------------------------------
           Num.
           gene     Num.        Obs.      Exp.
Locus#    copies   alleles      Het.       Het
-------------------------------------------------
     1        96         2   0.45833   0.44912
     2        96         6   0.41667   0.57785
     3        96         3   0.39583   0.65899
     4        96         2   0.33333   0.33333
     5        96         5   0.60417   0.68421
     6        96         3   0.60417   0.59671
     7        96         4   0.75000   0.71250
-------------------------------------------------
  Mean    96.000     3.571   0.50893   0.57325
  s.d.     0.000     1.512   0.14773   0.13699
-------------------------------------------------


================================
== Molecular diversity indices : (Torrijos)
================================

           Tajima, F., 1983.
           Tajima, F. 1993.
           Nei, M., 1987.
           Zouros, E., 1979.
           Ewens, W.J. 1972.
Sample size                                  : 96.00000
No. of haplotypes                            : 71

Allowed level of missing data                : 5.00000 %
Number of polymorphic loci                   : 7
Number of usable loci                        : 7


Average gene diversity over loci              :     0.573246 +/-     0.319867

(Standard deviations are for both sampling and stochastic processes)


================================================================================
== Summary of computations done within populations
================================================================================


----------------
Basic properties
-----------------------------------------------------------------------------------------------------------------------------------------------------------------------------------------------------------------------------------------------------------------------------------------------------------
          Statistics         Ambulong        Balanacan         Batangas    Brookes_Point       Cajidiocan          Calapan            Cawit           Lucena         Odiongan  Puerto_Princesa          Romblon            Roxas      San_Agustin         San_Jose         Torrijos        Mean        s.d.
-----------------------------------------------------------------------------------------------------------------------------------------------------------------------------------------------------------------------------------------------------------------------------------------------------------
  No. of gene copies              132               48              112               98              106               96               28              112               80               86               38               60              128               62               96      85.467      32.040
         No. of loci                7                7                7                7                7                7                7                7                7                7                7                7                7                7                7       7.000       0.000
  No. of usable loci                7                7                6                7                7                7                7                7                7                7                7                7                7                7                7       6.933       0.258
  No. of polym. loci                7                7                6                7                7                7                7                7                7                7                7                7                7                7                7       6.933       0.258
-----------------------------------------------------------------------------------------------------------------------------------------------------------------------------------------------------------------------------------------------------------------------------------------------------------


-----------------------
Expected heterozygosity
---------------------------------------------------------------------------------------------------------------------------------------------------------------------------------------------------------------------------------------------------------------------------------------------------------
Locus#         Ambulong        Balanacan         Batangas    Brookes_Point       Cajidiocan          Calapan            Cawit           Lucena         Odiongan  Puerto_Princesa          Romblon            Roxas      San_Agustin         San_Jose         Torrijos        Mean        s.d.   Tot. Het.
---------------------------------------------------------------------------------------------------------------------------------------------------------------------------------------------------------------------------------------------------------------------------------------------------------
     1          0.48531          0.54433          0.39591          0.40332          0.60323          0.50899          0.51587          0.42149          0.39462          0.35130          0.42817          0.46723          0.13349          0.55896          0.44912     0.44409     0.11099     0.46346
     2          0.64157          0.73316          0.71718          0.79508          0.70261          0.68520          0.76455          0.73472          0.74937          0.70889          0.84211          0.78701          0.78892          0.62031          0.57785     0.72323     0.07112     0.76831
     3          0.56269          0.63741          0.66120          0.57963          0.64888          0.64211          0.68783          0.67165          0.59462          0.66293          0.54623          0.62316          0.58994          0.62454          0.65899     0.62612     0.04253     0.66368
     4          0.44067          0.54167          0.47539          0.51147          0.35364          0.51513          0.44180          0.46412          0.51930          0.42107          0.55619          0.42712          0.26575          0.47401          0.33333     0.44938     0.08119     0.45731
     5          0.68980          0.78014          0.68919          0.73974          0.65211          0.63421          0.72751          0.60827          0.63829          0.72558          0.71266          0.77910          0.62857          0.61132          0.68421     0.68671     0.05699     0.72284
     6          0.56061          0.60638          0.64398          0.67894          0.60773          0.61294          0.57407          0.60135          0.67184          0.65554          0.56188          0.56328          0.59412          0.66896          0.59671     0.61322     0.04130     0.63508
     7          0.61080          0.71454          0.60167          0.60656          0.71716          0.68004          0.63228          0.68034          0.60570          0.72640          0.73400          0.73559          0.73142          0.70492          0.71250     0.67960     0.05289     0.71471
---------------------------------------------------------------------------------------------------------------------------------------------------------------------------------------------------------------------------------------------------------------------------------------------------------
  Mean          0.57021          0.65109          0.59779          0.61639          0.61219          0.61123          0.62056          0.59742          0.59625          0.60739          0.62589          0.62607          0.53317          0.60900          0.57325     0.60319     0.02804     0.63220
  s.d.          0.08678          0.09396          0.11868          0.13473          0.12185          0.07232          0.11684          0.11553          0.11372          0.15499          0.14163          0.14724          0.24221          0.07516          0.13699     0.12484     0.04111     0.12490
---------------------------------------------------------------------------------------------------------------------------------------------------------------------------------------------------------------------------------------------------------------------------------------------------------


----------------------------------------
Theta(H) under the infinite-allele model
---------------------------------------------------------------------------------------------------------------------------------------------------------------------------------------------------------------------------------------------------------------------------------------------
Locus#         Ambulong        Balanacan         Batangas    Brookes_Point       Cajidiocan          Calapan            Cawit           Lucena         Odiongan  Puerto_Princesa          Romblon            Roxas      San_Agustin         San_Jose         Torrijos        Mean        s.d.
---------------------------------------------------------------------------------------------------------------------------------------------------------------------------------------------------------------------------------------------------------------------------------------------
     1          0.94292          1.19455          0.65539          0.67595          1.52038          1.03662          1.06557          0.72859          0.65186          0.54154          0.74876          0.87699          0.15405          1.26739          0.81529     0.85839     0.33221
     2          1.78993          2.74751          2.53584          3.87988          2.36254          2.17660          3.24719          2.76956          2.98990          2.43515          5.33333          3.69496          3.73748          1.63370          1.36883     2.84683     1.02407
     3          1.28670          1.75795          1.95157          1.37888          1.84800          1.79412          2.20339          2.04557          1.46682          1.96672          1.20376          1.65367          1.43864          1.66338          1.93248     1.70611     0.29775
     4          0.78784          1.18182          0.90616          1.04694          0.54712          1.06242          0.79147          0.86611          1.08032          0.72732          1.25321          0.74556          0.36193          0.90118          0.50000     0.85063     0.25374
     5          2.22371          3.54839          2.21736          2.84236          1.87448          1.73381          2.66990          1.55277          1.76465          2.64407          2.48020          3.52685          1.69228          1.57279          2.16667     2.30069     0.65031
     6          1.27586          1.54054          1.80886          2.11468          1.54924          1.58357          1.34783          1.50847          2.04725          1.90310          1.28247          1.28978          1.46378          2.02077          1.47961     1.61439     0.29129
     7          1.56939          2.50311          1.51050          1.54171          2.53558          2.12543          1.71942          2.12833          1.53612          2.65500          2.75936          2.78205          2.72332          2.38889          2.47826     2.19710     0.49561
---------------------------------------------------------------------------------------------------------------------------------------------------------------------------------------------------------------------------------------------------------------------------------------------
Theta from
Mean H          1.32670          1.86606          1.48626          1.60683          1.57861          1.57222          1.63546          1.48399          1.47677          1.54704          1.67300          1.67429          1.14211          1.55756          1.34327     1.53134     0.17111
---------------------------------------------------------------------------------------------------------------------------------------------------------------------------------------------------------------------------------------------------------------------------------------------


-----------------
Number of alleles
---------------------------------------------------------------------------------------------------------------------------------------------------------------------------------------------------------------------------------------------------------------------------------------------------------
Locus#         Ambulong        Balanacan         Batangas    Brookes_Point       Cajidiocan          Calapan            Cawit           Lucena         Odiongan  Puerto_Princesa          Romblon            Roxas      San_Agustin         San_Jose         Torrijos        Mean        s.d. Tot. number
---------------------------------------------------------------------------------------------------------------------------------------------------------------------------------------------------------------------------------------------------------------------------------------------------------
     1                3                4                3                2                4                3                2                3                3                4                3                3                3                3                2       3.000       0.655           5
     2                5                5                7                6                6                7                5                7                6                7                9                7                9                5                6       6.467       1.302          11
     3                4                3                4                3                4                3                3                4                3                4                3                3                3                3                3       3.333       0.488           4
     4                4                3                3                3                2                3                3                3                3                3                3                2                2                3                2       2.800       0.561           4
     5                4                5                5                5                5                4                5                5                6                5                5                6                4                5                5       4.933       0.594           6
     6                3                3                4                4                5                3                3                4                3                3                3                4                3                3                3       3.400       0.632           6
     7                4                4                4                4                4                4                4                4                4                5                4                4                4                4                4       4.067       0.258           5
---------------------------------------------------------------------------------------------------------------------------------------------------------------------------------------------------------------------------------------------------------------------------------------------------------
  Mean            3.857            3.857            4.286            3.857            4.286            3.857            3.571            4.286            4.000            4.429            4.286            4.143            4.000            3.714            3.571       4.000       0.275       5.857
  s.d.            0.690            0.900            1.380            1.345            1.254            1.464            1.134            1.380            1.414            1.397            2.215            1.773            2.309            0.951            1.512       1.408       0.439       2.410
---------------------------------------------------------------------------------------------------------------------------------------------------------------------------------------------------------------------------------------------------------------------------------------------------------

===============================================================================
== GENETIC STRUCTURE ANALYSIS
===============================================================================


Number of usable loci for distance computation : 7
Allowed level of missing data                  : 0.050

List of usable loci :
---------------------
   1     2     3     4     5     6     7  

List of loci with too much missing data :
-----------------------------------------

NONE


======================================================================================
== Comparisons of pairs of population samples
======================================================================================

List of labels for population samples used below:
-------------------------------------------------
Label  	Population name
-----  	---------------
  1:	Ambulong
  2:	Balanacan
  3:	Batangas
  4:	Brookes_Point
  5:	Cajidiocan
  6:	Calapan
  7:	Cawit
  8:	Lucena
  9:	Odiongan
  10:	Puerto_Princesa
  11:	Romblon
  12:	Roxas
  13:	San_Agustin
  14:	San_Jose
  15:	Torrijos

------------------------
Population pairwise FSTs
------------------------


Distance method: Pairwise differences
                     1         2         3         4         5         6         7         8         9        10        11        12        13        14        15
           1   0.00000
           2   0.06006   0.00000
           3   0.05041   0.00868   0.00000
           4   0.11893   0.02900   0.06854   0.00000
           5   0.09918   0.04557   0.04985   0.08730   0.00000
           6   0.08137   0.01755   0.02378   0.07675   0.04059   0.00000
           7   0.07805   0.01826   0.06221   0.08407   0.07050   0.06084   0.00000
           8   0.05594   0.02224   0.00946   0.07467   0.04082   0.03740   0.07305   0.00000
           9   0.14770   0.04212   0.05142   0.07202   0.10605   0.06648   0.13145   0.06590   0.00000
          10   0.06346   0.02202   0.01568   0.05817   0.04697   0.05964   0.07642   0.02305   0.07845   0.00000
          11   0.02544   0.01990   0.02353   0.07451   0.07184   0.06152   0.04621   0.02242   0.08269   0.03209   0.00000
          12   0.04783   0.00675   0.00833   0.04784   0.04536   0.03724   0.03549   0.01750   0.07962   0.02444   0.00758   0.00000
          13   0.10391   0.05953   0.04923   0.07494   0.11891   0.11811   0.08738   0.06742   0.11895   0.06273   0.05245   0.02582   0.00000
          14   0.08095   0.02086   0.01997   0.09734   0.03213   0.02012   0.05456   0.02947   0.06712   0.03123   0.03252   0.03546   0.10596   0.00000
          15   0.10700   0.03986   0.03433   0.09380   0.06504   0.06176   0.05927   0.05177   0.10159   0.03684   0.05156   0.04720   0.08223   0.02437   0.00000


------------
FST P values
------------

Number of permutations : 10100

                       1                 2                 3                 4                 5                 6                 7                 8                 9                10                11                12                13                14                15
           1           *
           2   0.00000+-0.0000           *
           3   0.00000+-0.0000   0.08514+-0.0029           *
           4   0.00000+-0.0000   0.00030+-0.0002   0.00000+-0.0000           *
           5   0.00000+-0.0000   0.00000+-0.0000   0.00000+-0.0000   0.00000+-0.0000           *
           6   0.00000+-0.0000   0.01346+-0.0013   0.00010+-0.0001   0.00000+-0.0000   0.00000+-0.0000           *
           7   0.00000+-0.0000   0.05009+-0.0020   0.00000+-0.0000   0.00000+-0.0000   0.00000+-0.0000   0.00000+-0.0000           *
           8   0.00000+-0.0000   0.00069+-0.0003   0.01465+-0.0010   0.00000+-0.0000   0.00000+-0.0000   0.00000+-0.0000   0.00000+-0.0000           *
           9   0.00000+-0.0000   0.00020+-0.0001   0.00000+-0.0000   0.00000+-0.0000   0.00000+-0.0000   0.00000+-0.0000   0.00000+-0.0000   0.00000+-0.0000           *
          10   0.00000+-0.0000   0.00554+-0.0007   0.00426+-0.0006   0.00000+-0.0000   0.00000+-0.0000   0.00000+-0.0000   0.00000+-0.0000   0.00000+-0.0000   0.00000+-0.0000           *
          11   0.00802+-0.0009   0.03564+-0.0019   0.01218+-0.0011   0.00000+-0.0000   0.00000+-0.0000   0.00000+-0.0000   0.00772+-0.0008   0.00713+-0.0009   0.00010+-0.0001   0.00347+-0.0006           *
          12   0.00000+-0.0000   0.15870+-0.0036   0.08514+-0.0025   0.00000+-0.0000   0.00000+-0.0000   0.00000+-0.0000   0.01188+-0.0010   0.00386+-0.0006   0.00000+-0.0000   0.00208+-0.0005   0.23899+-0.0042           *
          13   0.00000+-0.0000   0.00000+-0.0000   0.00000+-0.0000   0.00000+-0.0000   0.00000+-0.0000   0.00000+-0.0000   0.00000+-0.0000   0.00000+-0.0000   0.00000+-0.0000   0.00000+-0.0000   0.00000+-0.0000   0.00079+-0.0003           *
          14   0.00000+-0.0000   0.01287+-0.0013   0.00287+-0.0006   0.00000+-0.0000   0.00000+-0.0000   0.00495+-0.0007   0.00030+-0.0002   0.00020+-0.0001   0.00000+-0.0000   0.00020+-0.0001   0.00743+-0.0008   0.00059+-0.0002   0.00000+-0.0000           *
          15   0.00000+-0.0000   0.00000+-0.0000   0.00000+-0.0000   0.00000+-0.0000   0.00000+-0.0000   0.00000+-0.0000   0.00030+-0.0002   0.00000+-0.0000   0.00000+-0.0000   0.00000+-0.0000   0.00020+-0.0001   0.00000+-0.0000   0.00000+-0.0000   0.00257+-0.0005           *


------------
Matrix of significant Fst P values
Significance Level=0.0500
------------

Number of permutations : 10100

                     1         2         3         4         5         6         7         8         9        10        11        12        13        14        15
           1                   +         +         +         +         +         +         +         +         +         +         +         +         +         +
           2         +                   -         +         +         +         -         +         +         +         +         -         +         +         +
           3         +         -                   +         +         +         +         +         +         +         +         -         +         +         +
           4         +         +         +                   +         +         +         +         +         +         +         +         +         +         +
           5         +         +         +         +                   +         +         +         +         +         +         +         +         +         +
           6         +         +         +         +         +                   +         +         +         +         +         +         +         +         +
           7         +         -         +         +         +         +                   +         +         +         +         +         +         +         +
           8         +         +         +         +         +         +         +                   +         +         +         +         +         +         +
           9         +         +         +         +         +         +         +         +                   +         +         +         +         +         +
          10         +         +         +         +         +         +         +         +         +                   +         +         +         +         +
          11         +         +         +         +         +         +         +         +         +         +                   -         +         +         +
          12         +         -         -         +         +         +         +         +         +         +         -                   +         +         +
          13         +         +         +         +         +         +         +         +         +         +         +         +                   +         +
          14         +         +         +         +         +         +         +         +         +         +         +         +         +                   +
          15         +         +         +         +         +         +         +         +         +         +         +         +         +         +          


---------------------------------------
Population average pairwise differences
----------------------------------------

Above diagonal    : Average number of pairwise differences between populations (PiXY)
Diagonal elements : Average number of pairwise differences within population (PiX)
Below diagonal    : Corrected average pairwise difference (PiXY-(PiX+PiY)/2)

Distance method: Pairwise differences
                       1          2          3          4          5          6          7          8          9         10         11         12         13         14         15
            1    3.99144    4.53535    4.24263    4.71003    4.59198    4.48366    4.50568    4.32826    4.78570    4.39843    4.28907    4.39179    4.26953    4.46933    4.48138
            2    0.26082    4.55762    4.34598    4.56611    4.62854    4.48003    4.53571    4.46522    4.55391    4.50145    4.56086    4.50000    4.34977    4.48757    4.45638
            3    0.21369    0.03395    4.06644    4.49818    4.39479    4.25930    4.47481    4.16358    4.34275    4.22488    4.32096    4.25833    4.06215    4.23301    4.18331
            4    0.55694    0.12993    0.30758    4.31475    4.71130    4.63850    4.72558    4.59074    4.57436    4.54794    4.69629    4.56650    4.30588    4.73585    4.59492
            5    0.45358    0.20705    0.21890    0.41125    4.28535    4.44841    4.63982    4.41375    4.73243    4.47905    4.66609    4.53884    4.50413    4.40079    4.43829
            6    0.36283    0.07611    0.10097    0.35602    0.18062    4.25022    4.57254    4.37974    4.51224    4.52059    4.59567    4.48229    4.47819    4.32863    4.40343
            7    0.33800    0.08494    0.26963    0.39625    0.32519    0.27548    4.34392    4.59279    4.89509    4.65033    4.57425    4.52440    4.35407    4.53341    4.43155
            8    0.24156    0.09543    0.03939    0.34239    0.18009    0.16366    0.32986    4.18195    4.47266    4.31613    4.37688    4.35714    4.20271    4.33468    4.32152
            9    0.70311    0.18823    0.22266    0.33012    0.50288    0.30026    0.63626    0.29481    4.17373    4.57151    4.65822    4.64646    4.43623    4.50524    4.55508
           10    0.27686    0.09679    0.06580    0.26471    0.21052    0.26963    0.35252    0.09930    0.35879    4.25171    4.45777    4.42461    4.21539    4.37903    4.28985
           11    0.10274    0.09143    0.09713    0.34831    0.33280    0.27995    0.21168    0.09529    0.38074    0.14131    4.38122    4.41535    4.22533    4.45034    4.41776
           12    0.20483    0.02995    0.03387    0.21788    0.20492    0.16594    0.16120    0.07493    0.36835    0.10751    0.03350    4.38249    4.12096    4.46586    4.40226
           13    0.44396    0.24111    0.19908    0.31866    0.53160    0.52323    0.35227    0.28188    0.51952    0.25969    0.20487    0.09987    3.65969    4.39957    4.17716
           14    0.35727    0.09241    0.08345    0.46213    0.14177    0.08718    0.24511    0.12736    0.30203    0.13684    0.14339    0.15828    0.45338    4.23268    4.22446
           15    0.47930    0.17121    0.14373    0.43119    0.28925    0.27196    0.25323    0.22419    0.46185    0.15764    0.22079    0.20465    0.34096    0.10176    4.01272


-----------
PXY P value
-----------

                         1           2           3           4           5           6           7           8           9          10          11          12          13          14

             2     0.00000
             3     0.00000     0.00030
             4     0.00000     0.00010     0.00000
             5     0.00000     0.00000     0.00000     0.00000
             6     0.00000     0.00198     0.00000     0.00000     0.00000
             7     0.00000     0.25198     0.00000     0.00000     0.00000     0.00020
             8     0.00000     0.00010     0.01465     0.00000     0.00000     0.00000     0.00020
             9     0.00000     0.00010     0.00000     0.00000     0.00000     0.00000     0.00000     0.00000
            10     0.00000     0.00069     0.00238     0.00000     0.00000     0.00000     0.00010     0.00000     0.00000
            11     0.00248     0.08307     0.00168     0.00000     0.00000     0.00010     0.01059     0.00792     0.00000     0.00673
            12     0.00000     0.08970     0.00663     0.00000     0.00000     0.00000     0.05099     0.00139     0.00000     0.00287     0.28990
            13     0.00000     0.00000     0.00000     0.00000     0.00000     0.00000     0.00000     0.00000     0.00000     0.00000     0.00000     0.00000
            14     0.00000     0.00307     0.00396     0.00000     0.00109     0.02000     0.00099     0.00059     0.00000     0.00198     0.00505     0.00040     0.00000
            15     0.00000     0.00000     0.00000     0.00000     0.00000     0.00000     0.00020     0.00000     0.00000     0.00000     0.00000     0.00000     0.00000     0.00228


---------------------
Corrected PXY P value
---------------------

                           1            2            3            4            5            6            7            8            9           10           11           12           13           14

              2      0.00000
              3      0.00000      0.10050
              4      0.00000      0.00050      0.00000
              5      0.00000      0.00000      0.00000      0.00000
              6      0.00000      0.01416      0.00010      0.00000      0.00000
              7      0.00000      0.04752      0.00020      0.00000      0.00000      0.00000
              8      0.00000      0.00089      0.01465      0.00000      0.00000      0.00000      0.00000
              9      0.00000      0.00020      0.00000      0.00000      0.00000      0.00000      0.00000      0.00000
             10      0.00000      0.00594      0.00426      0.00000      0.00000      0.00000      0.00000      0.00000      0.00000
             11      0.00980      0.03515      0.01386      0.00000      0.00000      0.00000      0.00802      0.00822      0.00020      0.00376
             12      0.00000      0.16000      0.09119      0.00000      0.00000      0.00000      0.01188      0.00426      0.00000      0.00228      0.23960
             13      0.00000      0.00000      0.00000      0.00000      0.00000      0.00000      0.00000      0.00000      0.00000      0.00000      0.00000      0.00079
             14      0.00000      0.01327      0.00297      0.00000      0.00000      0.00505      0.00040      0.00020      0.00000      0.00020      0.00762      0.00059      0.00000
             15      0.00000      0.00000      0.00000      0.00000      0.00000      0.00000      0.00030      0.00000      0.00000      0.00000      0.00020      0.00000      0.00000      0.00287


------------------------------------------------------------
Divergence times allowing for unequal population sizes (tau)
------------------------------------------------------------

Reference: Gaggiotti, O., and L. Excoffier, 2000.


----------
tau values
----------

                           1            2            3            4            5            6            7            8            9           10           11           12           13           14           15
              1      0.00000         
              2     -0.02316               0.00000         
              3      0.10486              -0.79243               0.00000         
              4      0.26249               0.00822               0.13066               0.00000         
              5      0.20777               0.05915               0.08287               0.21016               0.00000         
              6      0.16129              -0.11534               0.00868               0.18009               0.09038               0.00000         
              7      0.12481              -0.02467               0.10026               0.20229               0.16422               0.13579               0.00000         
              8      0.10325              -0.13508              -0.02259               0.16787               0.08342               0.07899               0.15773               0.00000         
              9      0.35950              -0.00375               0.10618               0.16038               0.25536               0.15023               0.32337               0.14992               0.00000         
             10      0.10922              -0.07189              -0.03218               0.13242               0.10599               0.13691               0.17664               0.04375               0.18090               0.00000         
             11     -0.13142               0.00318              -0.07825               0.17590               0.16593               0.13429               0.10626              -0.00444               0.17976               0.05617               0.00000         
             12      0.00911              -0.11173              -0.33741               0.10759               0.09776               0.07035               0.08016              -0.02951               0.17264               0.03401               0.01675               0.00000         
             13      0.19556              -0.28752              -0.00433              -0.00898               0.17733               0.18210               0.01002               0.02004               0.20083              -0.03868              -0.20982              -0.56315               0.00000         
             14      0.16122              -0.09556               0.00033               0.23518               0.06898               0.04347               0.11779               0.06159               0.15218               0.06870               0.05278               0.06185               0.13841               0.00000         
             15      0.24644              -0.12925               0.06995               0.19329               0.11398               0.11148               0.07308               0.09723               0.22981               0.03366               0.03365               0.01886               0.12674              -0.00854               0.00000         

An * denotes the cases where the estimation of divergence time did not converge


------------
tau P values
------------

                           1            2            3            4            5            6            7            8            9           10           11           12           13           14

              2      0.49604
              3      0.49484      0.51536
              4      0.46877      0.49338      0.49586
              5      0.45961      0.48633      0.48884      0.49157
              6      0.47686      0.51121      0.50993      0.50798      0.51460
              7      0.48509      0.48429      0.49328      0.47463      0.49109      0.48938
              8      0.50215      0.51220      0.51914      0.49365      0.49647      0.50984      0.48631
              9      0.49316      0.49185      0.50595      0.47560      0.50541      0.49591      0.45658      0.51270
             10      0.45697      0.48796      0.47532      0.48396      0.46500      0.48115      0.45404      0.48668      0.51193
             11      0.48605      0.47861      0.50075      0.48072      0.44866      0.51040      0.49181      0.50444      0.47011      0.46211
             12      0.48021      0.49185      0.51064      0.49309      0.48484      0.51682      0.48303      0.51534      0.51295      0.45977      0.48283
             13      0.48391      0.49278      0.49232      0.47002      0.44765      0.47798      0.47058      0.49610      0.49665      0.46032      0.47021      0.49086
             14      0.46655      0.49462      0.49865      0.49419      0.50571      0.52164      0.49671      0.50766      0.50040      0.46318      0.49590      0.48533      0.46171
             15      0.46452      0.46563      0.48151      0.46325      0.47214      0.47940      0.45970      0.47597      0.48507      0.43560      0.46359      0.45536      0.45524      0.46737


------------------------------------------------------------
Unequal population sizes (k)
------------------------------------------------------------

                           1            2            3            4            5            6            7            8            9           10           11           12           13           14           15
              1      0.00000         
              2     -0.03988               0.00000         
              3      0.41000               3.53247               0.00000         
              4      0.34600               0.96819               0.29205               0.00000         
              5      0.33010               0.83307               0.24532               0.51890               0.00000         
              6      0.31489               1.48358               0.04403               0.54718               0.54965               0.00000         
              7      0.23186               1.12552               0.23693               0.48058               0.45330               0.41232               0.00000         
              8      0.29799               1.45551              -0.22917               0.60072               0.64633               0.60622               0.62721               0.00000         
              9      0.42969               1.00945               0.37647               0.61075               0.55875               0.56592               0.57177               0.50727               0.00000         
             10      0.25899               1.27763              -0.19848               0.56135               0.54114               0.49856               0.56803               0.32262               0.44338               0.00000         
             11     -0.41982               0.98268              -0.29564               0.45039               0.42527               0.37943               0.45488              -0.02227               0.35816               0.26809               0.00000         
             12      0.02171               1.92556              -1.65878               0.42038               0.37886               0.29760               0.43909              -0.16409               0.35272               0.19354               0.49520               0.00000         
             13      0.69606               1.37149               1.01022               1.01281               0.80666               0.79449               0.98673               0.96546               0.75952               1.06462               1.33775               2.08470               0.00000         
             14      0.32473               1.36095               0.00191               0.54678               0.59438               0.55088               0.61653               0.39897               0.44947               0.53544               0.76178               0.73977               0.17345               0.00000         
             15      0.48811               1.27307               0.59504               0.68302               0.74187               0.72403               0.83255               0.69309               0.59192               0.88209               0.92051               0.95367               0.23298               1.03928               0.00000         

An * denotes the cases where the estimation of divergence time did not converge


------------
k P values
------------

                           1            2            3            4            5            6            7            8            9           10           11           12           13           14

              2      0.63690
              3      0.51868      0.14030
              4      0.53789      0.35520      0.53932
              5      0.53381      0.36398      0.54655      0.49485
              6      0.54663      0.27513      0.58377      0.48607      0.49990
              7      0.62001      0.34953      0.62497      0.60244      0.60619      0.60786
              8      0.53738      0.27189      0.61287      0.46913      0.45604      0.47246      0.39229
              9      0.53262      0.37806      0.54028      0.47939      0.49122      0.50150      0.41927      0.51110
             10      0.56115      0.30507      0.61621      0.49717      0.49638      0.50244      0.41067      0.55865      0.50408
             11      0.69501      0.36371      0.69715      0.56769      0.57915      0.59162      0.49451      0.66131      0.57819      0.60870
             12      0.60064      0.21863      0.79844      0.54182      0.55583      0.57316      0.46661      0.66295      0.56276      0.58180      0.46872
             13      0.48330      0.34774      0.42909      0.41666      0.46104      0.44474      0.37630      0.43424      0.43057      0.40586      0.33698      0.28840
             14      0.56136      0.27582      0.61397      0.51573      0.51445      0.51745      0.39880      0.55620      0.51945      0.51041      0.40419      0.43514      0.56697
             15      0.51632      0.31289      0.48811      0.46166      0.45376      0.44866      0.34310      0.47258      0.47379      0.44279      0.36097      0.39275      0.55298      0.38040


------------------------------------------------------------
Ancestral population sizes (theta0)
------------------------------------------------------------

                           1            2            3            4            5            6            7            8            9           10           11           12           13           14           15
              1      0.00000         
              2      4.55851               0.00000         
              3      4.13777               5.13842               0.00000         
              4      4.44755               4.55789               4.36752               0.00000         
              5      4.38421               4.56939               4.31192               4.50114               0.00000         
              6      4.32237               4.59538               4.25062               4.45841               4.35803               0.00000         
              7      4.38087               4.56038               4.37455               4.52329               4.47561               4.43675               0.00000         
              8      4.22501               4.60030               4.18617               4.42287               4.33033               4.30076               4.43506               0.00000         
              9      4.42620               4.55766               4.23657               4.41398               4.47707               4.36201               4.57172               4.32273               0.00000         
             10      4.28922               4.57334               4.25706               4.41551               4.37306               4.38369               4.47369               4.27238               4.39061               0.00000         
             11      4.42049               4.55768               4.39921               4.52039               4.50015               4.46137               4.46799               4.38132               4.47846               4.40160               0.00000         
             12      4.38269               4.61173               4.59574               4.45891               4.44107               4.41194               4.44424               4.38666               4.47382               4.39061               4.39860               0.00000         
             13      4.07397               4.63729               4.06649               4.31486               4.32680               4.29609               4.34405               4.18267               4.23540               4.25407               4.43515               4.68411               0.00000         
             14      4.30811               4.58312               4.23268               4.50066               4.33181               4.28516               4.41562               4.27308               4.35306               4.31033               4.39756               4.40401               4.26116               0.00000         
             15      4.23493               4.58563               4.11337               4.40163               4.32430               4.29195               4.35847               4.22429               4.32527               4.25619               4.38412               4.38340               4.05042               4.23300               0.00000         

An * denotes the cases where the estimation of divergence time did not converge


---------------
theta0 P values
---------------

                           1            2            3            4            5            6            7            8            9           10           11           12           13           14

              2      0.15852
              3      0.44375      0.04793
              4      0.33191      0.20478      0.34476
              5      0.37655      0.17205      0.36463      0.65305
              6      0.34110      0.13806      0.30077      0.60605      0.55741
              7      0.21282      0.27491      0.22066      0.26587      0.25371      0.26977
              8      0.36690      0.11597      0.30369      0.39855      0.39960      0.40157      0.23012
              9      0.35648      0.23298      0.39087      0.56552      0.65648      0.55648      0.30866      0.44756
             10      0.34978      0.16806      0.29207      0.62327      0.63489      0.61295      0.33833      0.41024      0.60845
             11      0.18428      0.31606      0.16154      0.32376      0.34928      0.27302      0.61422      0.21615      0.30376      0.36278
             12      0.23224      0.18373      0.09643      0.37832      0.36810      0.30321      0.44699      0.20896      0.33493      0.39009      0.50946
             13      0.40499      0.10989      0.26257      0.20723      0.29001      0.25786      0.17461      0.23116      0.24323      0.19188      0.13100      0.08816
             14      0.33423      0.14524      0.28600      0.61380      0.56787      0.48554      0.30828      0.35667      0.44077      0.59263      0.34251      0.40002      0.23709
             15      0.55534      0.13057      0.54227      0.39710      0.38760      0.37831      0.21400      0.42790      0.43764      0.38673      0.21954      0.25644      0.34466      0.29894

---------------------------------------------------
Matrix of coancestry coefficients as t/M=-ln(1-FST)
(M=N for haploid data, M=2N for diploid data)
---------------------------------------------------

Reference: Reynolds, J., Weir, B.S., and Cockerham, C.C. 1983.
                           1            2            3            4            5            6            7            8            9           10           11           12           13           14           15
              1     -0.00000
              2      0.06194     -0.00000
              3      0.05173      0.00872     -0.00000
              4      0.12662      0.02943      0.07100     -0.00000
              5      0.10445      0.04664      0.05114      0.09135     -0.00000
              6      0.08488      0.01770      0.02406      0.07985      0.04144     -0.00000
              7      0.08126      0.01843      0.06423      0.08782      0.07311      0.06277     -0.00000
              8      0.05757      0.02249      0.00951      0.07761      0.04168      0.03812      0.07585     -0.00000
              9      0.15981      0.04304      0.05279      0.07475      0.11211      0.06879      0.14094      0.06817     -0.00000
             10      0.06556      0.02227      0.01580      0.05993      0.04811      0.06150      0.07950      0.02332      0.08170     -0.00000
             11      0.02577      0.02010      0.02381      0.07743      0.07455      0.06349      0.04731      0.02267      0.08631      0.03262     -0.00000
             12      0.04902      0.00677      0.00837      0.04903      0.04642      0.03795      0.03614      0.01765      0.08297      0.02474      0.00761     -0.00000
             13      0.10972      0.06137      0.05048      0.07790      0.12659      0.12569      0.09143      0.06980      0.12665      0.06478      0.05388      0.02616     -0.00000
             14      0.08441      0.02108      0.02017      0.10241      0.03265      0.02032      0.05610      0.02992      0.06948      0.03173      0.03306      0.03610      0.11201     -0.00000
             15      0.11317      0.04067      0.03494      0.09850      0.06725      0.06375      0.06110      0.05316      0.10713      0.03753      0.05293      0.04835      0.08580      0.02468     -0.00000


----------------------------------------------------
Matrix of Slatkin linearized FSTs as t/M=FST/(1-FST)
(M=N for haploid data, M=2N for diploid data)
----------------------------------------------------

Reference: Slatkin, M., 1995. 
                           1            2            3            4            5            6            7            8            9           10           11           12           13           14           15
              1      0.00000
              2      0.06390      0.00000
              3      0.05309      0.00876      0.00000
              4      0.13499      0.02986      0.07358      0.00000
              5      0.11010      0.04774      0.05247      0.09565      0.00000
              6      0.08858      0.01786      0.02436      0.08312      0.04231      0.00000
              7      0.08465      0.01860      0.06634      0.09179      0.07585      0.06478      0.00000
              8      0.05926      0.02275      0.00955      0.08070      0.04256      0.03885      0.07880      0.00000
              9      0.17329      0.04398      0.05421      0.07761      0.11864      0.07121      0.15135      0.07055      0.00000
             10      0.06776      0.02252      0.01593      0.06176      0.04929      0.06343      0.08274      0.02360      0.08513      0.00000
             11      0.02610      0.02030      0.02410      0.08051      0.07740      0.06555      0.04845      0.02293      0.09014      0.03315      0.00000
             12      0.05024      0.00680      0.00840      0.05025      0.04751      0.03868      0.03680      0.01781      0.08651      0.02505      0.00764      0.00000
             13      0.11596      0.06329      0.05178      0.08101      0.13496      0.13393      0.09574      0.07229      0.13501      0.06693      0.05535      0.02651      0.00000
             14      0.08808      0.02130      0.02037      0.10784      0.03319      0.02053      0.05770      0.03037      0.07195      0.03223      0.03361      0.03676      0.11852      0.00000
             15      0.11982      0.04151      0.03555      0.10351      0.06957      0.06583      0.06301      0.05460      0.11308      0.03824      0.05436      0.04954      0.08959      0.02498      0.00000


------------------------------------------------------------------
Matrix of M values (M=Nm for haploid data, M=2Nm for diploid data)
------------------------------------------------------------------

                           1            2            3            4            5            6            7            8            9           10           11           12           13           14           15
              1
              2      7.82505
              3      9.41776     57.09369
              4      3.70402     16.74201      6.79504
              5      4.54130     10.47298      9.52917      5.22718
              6      5.64452     27.99579     20.52859      6.01506     11.81778
              7      5.90633     26.87801      7.53682      5.44732      6.59171      7.71844
              8      8.43798     21.97860     52.35271      6.19582     11.74829     12.86860      6.34497
              9      2.88532     11.36996      9.22380      6.44241      4.21457      7.02155      3.30358      7.08708
             10      7.37935     22.20166     31.39079      8.09584     10.14428      7.88296      6.04278     21.18847      5.87329
             11     19.15717     24.63060     20.75031      6.21059      6.46008      7.62809     10.32026     21.80487      5.54686     15.08101
             12      9.95292     73.56736     59.48957      9.95042     10.52348     12.92684     13.58673     28.07953      5.77975     19.96033     65.42227
             13      4.31171      7.89952      9.65676      6.17213      3.70492      3.73316      5.22231      6.91635      3.70331      7.47075      9.03293     18.86196
             14      5.67683     23.46969     24.54203      4.63649     15.06400     24.35119      8.66486     16.46446      6.94941     15.51154     14.87587     13.60033      4.21872
             15      4.17292     12.04446     14.06343      4.83043      7.18721      7.59574      7.93543      9.15803      4.42170     13.07377      9.19806     10.09240      5.58077     20.01442


////////////////////////////////////////////////////////////////////
END OF RUN NUMBER 1 (15/01/15 at 12:21:42))
Total computing time for this run : 0h 7m 20s 889 ms
////////////////////////////////////////////////////////////////////
```
